# Microfiber knot resonator with 10^7^ Q-factor record

**DOI:** 10.1038/s41377-025-02124-1

**Published:** 2026-03-06

**Authors:** Xinxin Zhou, Zixuan Ding, Fei Xu

**Affiliations:** 1 National Laboratory of Solid-State Microstructures and College of Engineering and Applied Sciences, Nanjing University, Nanjing, 210023 China; 2 Shenzhen Research Institute of Nanjing University, Shenzhen, 581000 China

**Keywords:** Microresonators, Fibre optics and optical communications

## Abstract

Despite the realization of ultra-high-quality-factor (UHQ) in various dielectric micro-resonators with extensive applications, UHQ microfiber resonators which are directly compatible with all-fiber framework have not yet been achieved, primarily because of the insufficient research on the mechanical properties of microfibers, and the challenges of coupling regulation. Here, we constructed an UHQ microfiber knot resonator (MKR) fabrication model, addressing the decades-long Q-factor bottleneck and achieving a record Q-factor of 3.9 × 10^7^, which is an improvement of three orders of magnitude compared to conventional levels. By controlling environmental parameters for producing high-quality microfibers with uniform stress and low loss, along with experimental and theoretical investigation in coupling mechanism, optimized conditions are attained, offering experimental guidance for fabricating UHQ-MKR stably and reproducibly. After fabrication and characterization, the UHQ-MKR device is also applied into an all-fiber laser scheme to boost narrow-linewidth single-frequency laser operation, highlighting the potential of the resonator. The research opens an era of UHQ microfiber resonator exceeding 10^7^ level, paving the path for more precision and efficient microfiber guiding-wave photonics.

## Abstract (web summary)

We constructed a silica microfiber knot resonator fabrication model and achieved a record high Q-factor of 3.9 × 10^7^ for this intriguing all-fiber soft resonator scheme, paving the path for more precision and efficient microfiber guiding-wave photonics.

## Graphical abstract

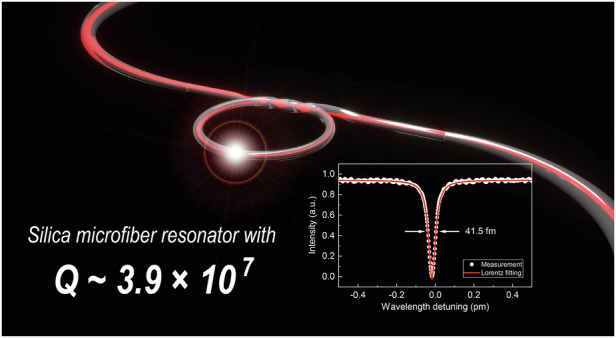

## Introduction

As fundamental photonic devices, high-quality-factor (high-Q) optical resonators significantly enhance the light-matter interactions, forming the cornerstones of scientific advances in nonlinear optics^1^, quantum physics^2^, high-sensitivity sensing^3^ and other fields^4^. Over the past decades, a variety of high-Q microcavities have emerged^5^, including Fabry-Pérot cavities^6^, whispering-gallery-mode (WGM) microcavities^7^, and photonic crystal microcavities^8^. Among these, WGM cavities such as microspheres or microtoroids have long been celebrated for their ultra-high Q-factors (UHQ) up to 10^8^ or beyond. However, despite the remarkable optical qualities, these free-standing WGM cavities face practical challenges in terms of mechanical stability, integration, and packaging.

With the advancement of micro-fabrication techniques and semiconductor processes, waveguide microcavities are gradually emerging as a highly promising technology due to their compact size and high integration with other photonic devices. Yet, inherent material properties and fabrication limits in polishing and etching process typically restrict their Q-factors to 10^6^ level^9–12^, with fabrication processes remaining costly. The geometrical mismatch between on-chip waveguide and fiber modes also poses significant coupling loss for achieving efficient connection in application.

In parallel, microfiber-based resonators, the waveguide cavities fabricated directly from tapered optical fibers, have emerged as a complementary platform exhibiting distinctive advantages. These microfiber resonators are inherently flexible, monolithic, and self-supported structures with excellent compatibility to all-fiber systems, resulting in low coupling loss and simplified packaging^13–15^. Unlike bulky or on-chip microcavities, microfiber resonators feature mechanically bound coupling and free-standing waveguide geometry, leading to high flexibility and robustness that uniquely enable applications in emerging areas like wearable health monitoring^16^ or underwater acoustics^17^.

Typical microfiber resonators can be configured as microfiber loop resonators (MLR)^18,19^, microfiber coil resonators (MCR)^20–22^, or microfiber knot resonators (MKR)^23–25^. Theoretically, UHQ approaching 10^9^ is possible given the low loss and smooth surface quality achievable by flame-brushing fabrication techniques^26^. Whereas, historically, microfiber resonators have suffered from relatively low experimental Q-factors around 10^4^ ~ 10^5^ level due to non-standard workflow^14,18–25,27–43^. This has led to a general perception that microfiber resonators are inherently limited in Q, restricting their use in more demanding photonic applications.

In this work, we present the first systematic investigation into the long-standing Q-factor bottleneck of microfiber knot resonators, identifying and quantifying the fundamental limiting factors of microfiber mechanical properties and precise coupling control in the knot region. The optimal coupling parameter conditions for achieving UHQ are derived, and the influence of environmental temperature and humidity on microfiber mechanical stability gets revealed. Guided by these insights, we develop an environment-tailored fabrication workflow that elevates the Q-factor to 3.9 × 10^7^, representing nearly a three-orders-of-magnitude improvement over conventional values. The fabricated UHQ-MKRs exhibit thermal bistability and well-defined polarization characteristics, and their application in the all-fiber laser scheme which enables single-frequency operation is also demonstrated, highlighting the value of Q-factor exceeding 10^7^.

This leap not only redefines the practical capabilities of MKRs, but also opens new research avenues in cavity-enhanced sensing or flexible optomechanical systems, where the unique combination of UHQ, mechanical robustness, and structural flexibility can enable functionalities beyond those of classical WGM microcavities.

## Results

### Ultra-high-Q microfiber knot resonator

**Fig. 1:**
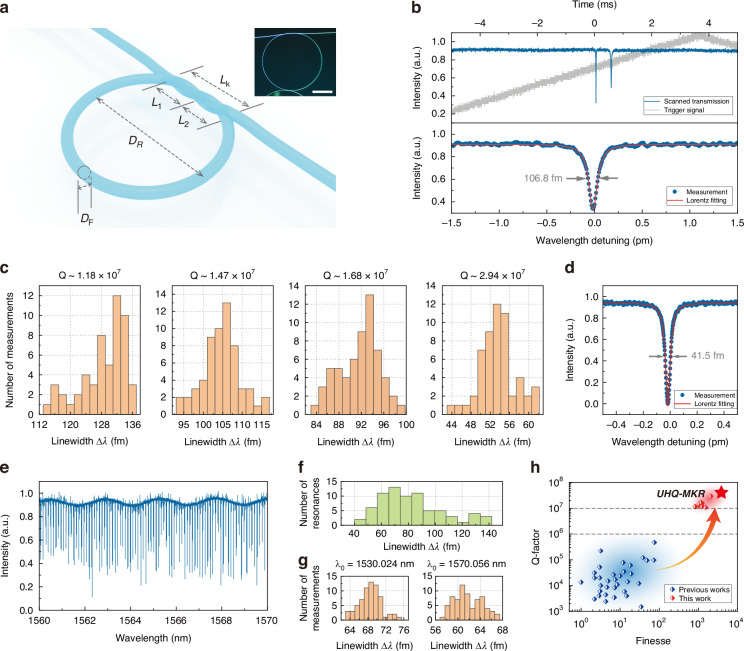
The ultra-high-Q microfiber knot resonator. **a** Schematic diagram of a MKR, where ${D}_{{\rm{R}}}$ is the diameter of ring section, ${D}_{{\rm{F}}}$ is the cross-section diameter of microfiber, ${L}_{{\rm{k}}}$ is the length of knot coupling region, while ${L}_{1}$ and ${L}_{2}$ are the length of two sub-knot areas, respectively. Inset shows microscopic photos of typical fabricated MKR, where scale bar represents 500 μm. **b** Fine transmission spectrum obtained by the wavelength detuning scan. Local zoom-in in the lower plot shows a standard Lorentz resonance mode around 1560 nm with Q-factor exceeding 1.4 × 10^7^. **c** Histograms of multiple measurement results for resonance linewidth of UHQ-MKR samples with varied Q-factors exceeding 10^7^. **d** The transmission spectrum of UHQ-MKR with largest Q-factor obtained in experiment which is near 3.9 × 10^7^. **e** 10 nm-band transmission spectrum scanning for UHQ-MKR, with **f** the corresponding statistic for linewidths of recorded resonances, and (**g**) the histograms of multiple measurement results for linewidths of resonances located around 1530 nm and 1570 nm respectively. **h** Q-factor and finesse summary of microfiber resonators, where the performance of microfiber resonators reported in previous literatures are gathered, including MKR, MLR, and MCR^14,18–25,27–43^. The experimental record of this work is represented by the red star

The MKR is fabricated by winding a single microfiber, with its basic structure shown in Fig. 1a. The MKR consists of two parts: the knot coupling region and the ring region. The knot region is integrated through mechanical stress, providing structural stability and robustness. As denoted, *L*_*k*_ is the length of the knot coupling region, while *L*_1_ and *L*_2_ are the lengths of the two sub-knot twisting areas, respectively. The diameter of the ring region is *D*_R_, and the microfiber has a cross-section diameter of *D*_F_. The inset in Fig. 1a shows microscopic photos of typical fabricated MKR, demonstrating a smooth surface of the microfiber, along with a robust and stable structure. The fundamental fabrication flow of MKR involves 3 main steps: microfiber tapering, knotting and coupling tuning, summarized as “TKT” method. The key to effectuate UHQ lies with the two “T” procedures, tapering and tuning, and detailed description can be found in Methods. The Q-factor is a crucial parameter of the microfiber resonator, primarily determined by the coupling in the knot region and the loss in the microfiber waveguide. To obtain UHQ samples, precise control of the waist diameter, coupling length, and micro-ring diameter is essential, which introduces significant challenges to the fabrication process.

Due to the resolution limitations of the optical spectrum analyzer (20 pm), the transmission spectrum data becomes inaccurate when the MKR’s Q-factor exceeds 10^5^. Thus we introduce the detuning-scan method into the research of microfiber resonators, which enables real-time measurement of transmission spectrum and Q-factor for MKR samples to determine the critical coupling position where the resonator reaches UHQ (see Methods). As shown in Fig. 1b, the detuning-scan Q-factor measurement system displays a typical resonance spectrum for the UHQ-MKR sample at a central wavelength of 1560 nm, with a full width at half maximum (FWHM) reaching 0.11 pm for a single resonance. The corresponding Q for this linewidth is 1.46 × 10^7^. To ensure the accuracy of measurement results, we typically perform >50 repeated scans on a single resonance mode to eliminate random measurement errors. Figure 1c presents statistical histograms of the resonance linewidth obtained from multiple measurements of a single resonance mode in different samples. In our experiments, we obtained UHQ-MKR samples with Q ranging from 1 × 10^7^ to nearly 4 × 10^7^. It can be observed that the fluctuation range of the measured resonance linewidth does not exceed 30 fm, with a maximum standard deviation of 5.79 fm and a minimum of 3.56 fm, showing a relatively concentrated distribution. The largest Q-factor of MKR obtained in our experiment is shown in Fig. 1d, with FWHM ~ 0.04 pm and Q ~ 3.9 × 10^7^. Although some fiber cavity based on directional fiber coupler and large-scale fiber can also achieve ultra-high Q-factors with extremely small FSR, their larger mode volumes and lower energy densities make such macroscopic resonators unable to replace microcavities in many applications^44^.

It should be noted that the above measurement results are based on resonant modes near a wavelength of 1560 nm. In addition, we also characterized the broadband resonance properties of UHQ-MKR. Considering the oscilloscope’s acquisition precision limitations, the single-scan bandwidth was set to 10 nm, as shown in Fig. 1e. By extracting and fitting the resonant modes recorded within this range, we obtained the linewidth statistical distribution shown in Fig. 1f. The results indicate that >57% of the resonant modes have a Q-factor >2 × 10^7^, while the Q-factors of all modes exceed 1.1 × 10^7^. Furthermore, we conducted multiple measurements on the resonant modes around 1530 nm and 1570 nm respectively, with histograms presented in Fig. 1g. At approximately 1530 nm, the sample’s Q-factor reached 2.23 × 10^7^, with a linewidth fluctuation standard deviation of 2.48 fm. Around 1570 nm, the Q-factor was 2.56 × 10^7^, with a linewidth fluctuation standard deviation of 2.52 fm. The test results demonstrate that the UHQ-MKR samples could maintain a high-Q-factor feature exceeding 10^7^over a relatively wide wavelength range. Compared to previously reported microfiber resonators (Fig. 1h), the achieved Q-factor has been improved dramatically by orders of magnitude, marking the highest record ever in the field of microfiber resonators. To obtain more accurate characterization of the intrinsic optical performance of the resonator, the cavity finesse which is directly related to optical loss and independent of cavity length also gets compared. As in Fig. 1h, the device also exhibits the highest finesse for microfiber resonators, representing an ~30-fold improvement over conventional ones.

As aforementioned, quality management of microfiber and elaborate coupling tuning are keys to the high Q-factor. Therefore, it is essential to take deep investigation into the two crucial factors to build more complicated fabrication model of UHQ-MKR.

### Quality management of microfiber

Since the silica microfiber employed in the UHQ-MKR fabrication process is prepared through flame-brushing method where the oxyhydrogen flame serves as heat source, the quality of microfiber is sensitive to environment. Chiefly, the environmental temperature and humidity influence the mechanical properties of the microfiber by affecting thermal field distribution, flame stability, and the melting temperature of silica fiber, which ultimately impacts the Q-factor of the MKR. The optimal quality microfiber is characterized by superior mechanical properties and lower losses (loss analysis in Supplementary Note 2). We determined the optimal environment through statistical analysis, which requires simultaneously meeting two conditions: first, the initial temperature and humidity during the fabrication process correspond within a certain range; second, the humidity remains constant. During the experiment, the silica fiber with melting temperature of 1700 °C is positioned at various locations within the thermal field of the oxyhydrogen flame, resulting in microfibers with different mechanical properties. The first condition requires that the optical fiber is positioned at a specific location within the flame core region, balancing the molten state with the material’s microstructure, which reduces internal stress and minimizes defects. The second condition is to ensure the stability of the thermal field. Fluctuations in the thermal field lead to instability in the molten state, which may induce uneven stress distribution within the fiber and result in the formation of inhomogeneous structures. Moreover, regions of stress concentration are prone to becoming initiation points for brittle fracture, which can also reduce the toughness and impact resistance of the microfiber. The internal stress distribution can be indirectly reflected by testing the breaking points of the microfibers, since it is challenging to directly measure the internal stress within the micrometer-scale silica fibers.

**Fig. 2:**
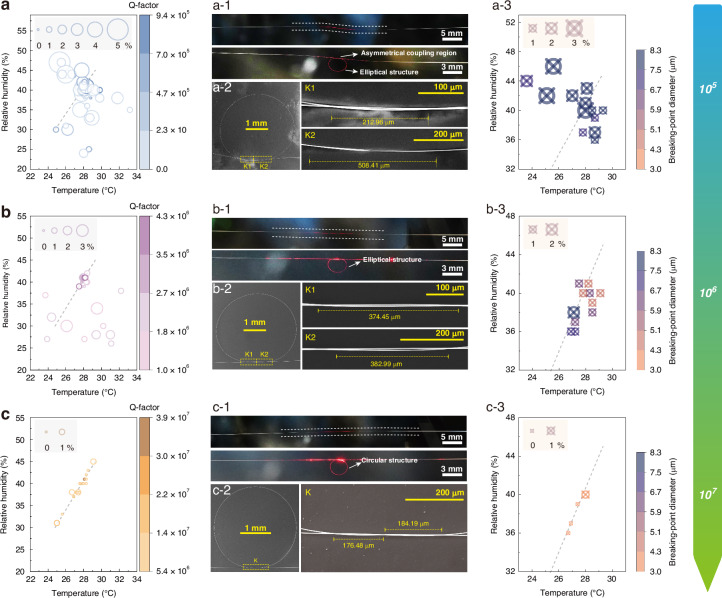
Optimization of environmental parameters for UHQ-MKR. The temperature and humidity dependent distribution for samples with **a** ${{\rm{Q}}}_{1} < {10}^{6}$, **b** ${{10}^{6} < {\rm{Q}}}_{2} < {5\times 10}^{6}$, and **c** ${{\rm{Q}}}_{3} > {5\times 10}^{6}$. The size of circle for each data point represents humidity fluctuation during fabrication process, with larger circle represents severe fluctuation. The dashed line stands for the optimized temperature-humidity condition derived from experiments. Sub-levels of Q-factor values are denoted with different color depth. **a**-**1**, **b**-**1**, **c**-**1** Photos of typical microfiber that leads to Q within the corresponding range **(**upper) and the formed MKR sample (lower). **a**-**2**, **b**-**2**, **c**-**2** SEM photo of sample with Q within the corresponding range, where the insets are the local scanning of knot coupling areas. **a-3**, **b**-**3**, **c**-**3** Statistical results of cross-section diameter at breaking-point position for microfibers that leads to Q within the corresponding range. The size of icons for each data point also represents humidity fluctuation. Here the diameter of the waist region of the microfiber is ~3 μm

The MKR forming mechanism depending on mechanical properties of the microfiber was studied by controlling the environmental temperature and humidity. Figure 2 demonstrates the temperature and humidity dependent distribution for samples with (a) ${{\rm{Q}}}_{1} < {10}^{6}$, (b) ${{10}^{6} < {\rm{Q}}}_{2} < {5\times 10}^{6}$, and (c) ${{\rm{Q}}}_{3} > {5\times 10}^{6}$. The size of circle for each data point represents humidity fluctuation during fabrication process (circle diameter proportional to the fluctuation). The dashed line represents the optimal temperature-humidity conditions derived from experiments. The optical microscope and scanning electron microscope (SEM) photos in Fig. 2 were obtained using samples of similar dimensions, with a FSR of ~0.14 nm. For all the samples collected in Fig. 2, the fabrication process of the microfiber was conducted under conditions where the flow rate was kept constant, while the distance between the flame nozzle and the fiber being processed remained fixed, ensuring that the only experimental variables were the environmental temperature and humidity. Before the preparation of the microfiber, the optical fiber is placed at a specific location within the flame core region as a calibration step. Identical parameters were adopted in tapering process to ensure waist diameter around 3 μm for each microfiber.

**Fig. 3:**
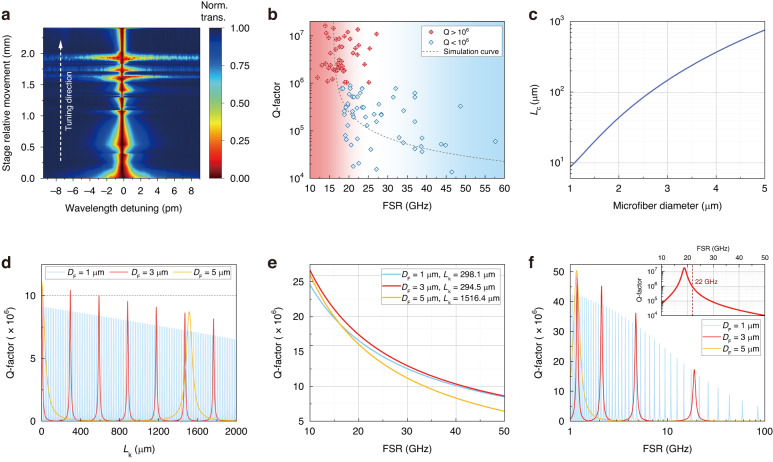
Coupling tuning in MKR-fabrication process. **a** The evolution map of single resonance mode spectrum when the step-motor stage keeps moving in experiment. **b** Q-factor distribution versus FSR of MKR in experiment. **c** Calculated coupling length of two adjoined microfiber waveguides ${L}_{{\rm{c}}}$ with varied cross-section diameter. **d** Simulated Q-factor distribution versus ${L}_{{\rm{k}}}$ for varied microfiber diameters, where the size of MKR ring is fixed. **e** Simulated Q-factor distribution versus FSR for varied microfiber diameters, where the ${L}_{{\rm{k}}}$ is fixed for each condition. **f** Simulated Q-factor distribution versus FSR for varied microfiber diameters under “standard knot” condition, where ${L}_{{\rm{k}}}$ varies correspondingly when MKR ring shrinks

In Fig. 2a, the fabrication environment features humidity fluctuations ≥2% or deviations from the optimal temperature-humidity baseline, represents a non-ideal environment, under which the ${{\rm{Q}}}_{1} < {10}^{6}$. We captured corresponding photos of the microfiber in a suspended state at the end of the fabrication process. The twisted and irregular structure of the microfiber indicates the presence of significant internal stress (see the upper panel of Fig. 2a-1), which also results in the asymmetric distribution in the MKR’s knot coupling region, making it difficult to achieve ideal coupling and leading to a lower Q value (the lower panel of Fig. 2a-1) and Fig. 2a-2). As shown in Fig. 2a-3, the fracture locations of the microfibers prepared in such environments get tested, and the results indicate that most breakage points occurred at a diameter of around 8 μm, rather than at the thinnest waist region of 3 μm. This also suggests the presence of internal stress in the microfibers, while stress concentration region serves as fracture point. Additional fracture characterizations can be found in Supplementary Note 3.

Figure 2b corresponds to an environment with humidity fluctuations of ~1% deviations from the optimal baseline, represents a non-ideal environment where the ${{10}^{6} < {\rm{Q}}}_{2} < {5\times 10}^{6}$. Compared to the microfibers in the ${{\rm{Q}}}_{1}$ state, those in the ${{\rm{Q}}}_{2}$ state show a slight collapse, with stress distribution uniformity determined to lie between ${{\rm{Q}}}_{1}$ and ${{\rm{Q}}}_{3}$, as shown in the upper part of Fig. 2b-1. In this state, the microfiber does not exhibit any significant structural irregularities, and the knot region of the MKR is symmetrically distributed, indicating that MKRs based on this type of microfiber have the potential to achieve ideal coupling conditions (Fig. 2b-2). However, the loop region of the MKR is typically flattened into an elliptical shape, inducing a larger coupling region length for the same FSR (757.44 μm in this example) (see the lower part of Fig. 2b-1 and Fig. 2b-2). Constrained by the coupling length in the knot region, ${{\rm{Q}}}_{2}$ can exceed 1 × 10^6^ but remains limited to below 5 × 10^6^. The coupling length in the knot region is an important factor affecting the Q-factor, and the detailed theoretical analysis will be discussed in the next section. From Fig. 2b-3 it can be concluded that microfibers of this state tend to break at locations with cross-section diameter around 6 μm, which are closer to waist area and implying subdued stress unevenness.

The ideal environment for microfiber fabrication is summarized as constant humidity and temperature-humidity levels close to the baseline, which contributes to ${{\rm{Q}}}_{3}$ ranging from 5 × 10^6^ to 3.9 × 10^7^, as shown in Fig. 2c. At the end of the fabrication process, the microfiber displays a uniformly arched state, indicating a uniform stress distribution. As shown in Fig. 2c-1 and Fig. 2c-2, the knot region of the MKR is symmetrically distributed, and the ring section is circular, demonstrating a shorter coupling region length for the same FSR (360.67 μm in this example) compared to Q_2_. This coupling region length falls within the ideal coupling range, and optimal coupling can be achieved through fine adjustments. Among these samples, over 50% achieved Q > 10^7^. As can be seen from Fig. 2c-3, the fracture point of microfibers prepared here situated in its thinnest waist region, showing balanced residual stress distribution.

Quality management of microfibers is a prerequisite for achieving UHQ-MKR. In other words, only by fabricating high-quality microfibers, and then applying precise coupling tuning, can UHQ-MKRs be obtained. It should be noted that there still exists room between our record and the theoretical limitation (10^9^). The current limiting factors and possible optimization strategies for microfiber qualities are discussed in Supplementary Note 2.

### Coupling tuning

To achieve a more comprehensive understanding of another crucial prerequisite to UHQ-MKR, the coupling tuning process, the spectral evolution of MKR’s resonance with the gradually pulling of motorized stage was also recorded and mapped, shown in Fig. 3a. Here the MKR was formed by microfiber with waist diameter of 3 µm. The length of the knot area shows an irregular decreasing trend during the tuning process, with a variation range of 200−500 µm. The spectral FWHM exhibits approximately periodic fluctuations but follows an overall trend of decreasing. The highest Q-factor achieved by this sample is 1.17 × 10^7^ when the stage relative movement is 1.32 mm. The mapping indicates that the tuning of microfiber knot should be elaborate enough to catch the periodically emerging UHQ point.

Since the movement of motorized stage reduces the ring size of MKR simultaneously, the Q-factor distribution versus cavity FSR was also investigated, as illustrated in Fig. 3b. In the experiment, the samples formed by microfiber with a diameter of 3 µm show a trend of gradually decreasing Q value with decreasing size of the ring section, i.e. increasing FSR. The experimental results demonstrate that for samples with a Q-factor >10^6^, the FSR are mostly located within 0.16 nm, or less than 20 GHz, which could be an empirical parameter for ring-size controlling to achieve UHQ.

In order to theoretically interpret the experimental results, here we adopt the coupled mode theory for numerical model building of two adjoined microfiber waveguides. In the MKR, the microfibers are brought into close proximity at the knot region, forming a coupling zone where the optical field undergoes periodic energy exchange. This periodic transfer arises from repeated coupling and decoupling of the optical power between the two waveguides. The coupling length *L*_*c*_ serves as the key parameter that defines this transfer cycle, representing the distance over which light is fully coupled from one waveguide to the other. The theoretical value of *L*_*c*_ varying with different microfiber diameters in UHQ-MKR is investigated at wavelength of 1550 nm (detailed derivation in Supplementary Note 4). As shown in Fig. 3c, the coupling length increases with an increasing microfiber diameter. The coupling coefficient *κ* is related to the coupling length *L*_*c*_ by the expression $\kappa =\pi / (2{L}_{{\rm{c}}})$. A narrower microfiber exhibits a stronger evanescent field, leading to stronger coupling which means more frequent energy exchange and thus a shorter *L*_*c*_. The coupling state determined by *L*_*c*_ will lead to specific resonance behavior and ultimately affect the achievable Q-factor.

Based on the calculated coupling coefficients *κ*, the Q-factor of MKR under certain loss condition can be obtained through ring-resonator model where the transmission is derived as

1 $$T=\left(1-\gamma \right){\left|\frac{\cos \left(\kappa {L}_{{\rm{k}}}\right)-\sqrt{1-\gamma }\exp \left(-\frac{\rho }{2}{L}_{{\rm{r}}}-i\beta {L}_{{\rm{r}}}\right)}{1-\sqrt{1-\gamma }\cos \left(\kappa {L}_{{\rm{k}}}\right)\exp \left(-\frac{\rho }{2}{L}_{{\rm{r}}}-i\beta {L}_{{\rm{r}}}\right)}\right|}^{2}$$

where *L*_r_ is the length of ring section, *γ* and *ρ* denote the overall insertion loss coefficient and the attenuation coefficient of the ring section, respectively.

Firstly, the whole size of MKR (${{L}_{{\rm{k}}}+L}_{{\rm{r}}}$) gets fixed and the dependence between Q-factor and coupling region length *L*_k_ is investigated for microfiber diameters of 1, 3, and 5 μm. The loss coefficients are set according to experiments. As shown in Fig. 3d, the Q-factor exhibits periodic fluctuations with varied coupling region length, which originates from the phase-matching conditions of evanescent-wave coupling between two identical waveguides. As aforementioned, optical power undergoes periodic transfer between the evanescent identical waveguides with a period of 2*L*_c_. For the entire MKR structure, the critical coupling condition must be satisfied so as to precisely enable the resonator to store the maximum energy, thereby realizing high Q. For samples with a microfiber diameter of 1 μm, the fluctuation period is shorter, indicating that more *L*_k_ values can achieve high Q. However, the linewidth of the periodic high-Q peaks is correspondingly much narrower, slight offset from the peak will cause the Q-factor to drop dramatically, hindering the possibility to attain high Q values in experiments as the precision of the motorized displacement stage is limited. For samples with a diameter of 3 μm, the linewidth of the high-Q peaks is over 15 μm, and the current stepper motor with a resolution of 10μm is possible to access the high-Q regime. To reliably and precisely pinpoint the maximum Q position in experiments, higher-precision tuning methods, such as sub-micron displacement stages, may also be considered. In addition, the peak Q-factor value also decreases with increasing *L*_k_, which is universal regardless of microfiber diameter. For samples with a microfiber diameter of 5 μm, simulations indicate that a Q-factor exceeding 10^7^ is only achievable when the *L*_k_ is below 100 µm, making it extremely challenging to achieve experimentally. For the next peak with large *L*_k_, the peak Q value has dropped to a relatively lower level. This simulation could explain why microfiber with a diameter of 3 µm was selected for preparing UHQ-MKR after trials: it was a moderate choice that favoring *Q* > 10^7^ with less difficulty.

Secondly, ${L}_{{\rm{k}}}$ is fixed, while the relationship between Q-factor and MKR’s size, i.e. the FSR, is investigated. We selected the coupling region length corresponding to the maximum Q value in Fig. 3d as a reference. For example, for a microfiber with a diameter of 3 µm, ${L}_{{\rm{k}}}$ was set to 294.5 µm. The Q values all exhibited a decreasing trend as the size of MKR decreased, as depicted in Fig. 3e, corresponding to the trend observed in Fig. 3b. The primary reason for the decrease in Q-factor with increasing FSR is not the increased microfiber bending loss caused by a larger FSR (smaller ring), as explained in detail in Supplementary Note 3. When the ring size is reduced while keeping the coupling region length constant, photons traverse the coupling region more frequently. Given that the loss in the coupling region is fixed, more frequent passes through this region increase the loss per unit time, shortening the photon lifetime and reducing the Q-factor.

Finally, to more accurately simulate the experimental conditions, we propose a “standard knot” geometrical model to describe the coupling tuning process during UHQ-MKR’s fabrication. As afore discussed, the ideal microfiber for achieving UHQ would lead to a knot with perfect circular ring section, named as “standard knot”. In such case, considering that the microfiber knot is tight, the coupling region length ${L}_{{\rm{k}}}$, the microfiber diameter ${D}_{{\rm{F}}}$, and the ring section radius $R={D}_{{\rm{R}}}/2$ satisfy the approximate geometrical relation ${L}_{{\rm{k}}}=4\sqrt{{D}_{{\rm{F}}}\left(R-{D}_{{\rm{F}}}\right)}$ (see Supplementary Note 4 for details). This relation corresponds to the coupling tuning scenario in experiment where the size of MKR and its coupling area change simultaneously. Thereby, the relationship between Q-factor and FSR was simulated as shown in Fig. 3f. The theoretical results indicate that for samples prepared with 3 µm microfiber, the Q-factor would exceed 10^6^ when the FSR is <22 GHz, which shows proximity to the experimental results. We also plot the simulated curve in Fig. 3b for fitting experimental data (gray dashed line).

### Characterization of UHQ-MKR

**Fig. 4:**
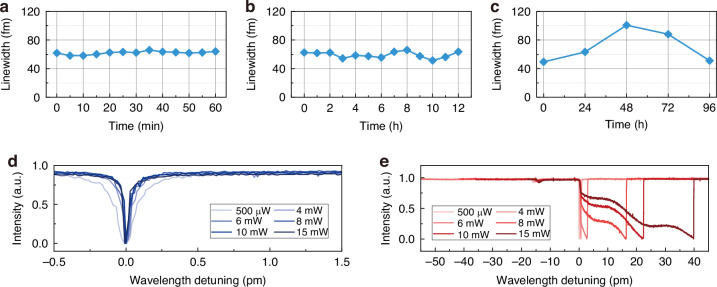
Long-term stability and thermal effects of UHQ-MKR. **a** 1 h, (**b**) 12 h, and (**c**) 96 h stability of the resonant mode linewidth for UHQ-MKR sample. **d** Measured resonances for increasing input power with blue-detuning. **e** Measured resonances for increasing input power with red-detuning

For the prepared UHQ-MKR samples, we first characterized their stabilities to testify how long the Q-factor exceeding 10^7^ can be maintained. We suspended the sample in an acrylic enclosure to prevent airflow disturbances and dust contamination. The linewidths of the resonant modes were continuously monitored to assess stability over both short and long periods. As shown in Fig. 4a, within a 1 h period, the device maintained high stability, with the linewidth consistently around 60 fm (corresponding to a Q-factor of ~2.6 × 10^7^) and a fluctuation standard deviation of only 2.18 fm. Furthermore, within a 12 h period, the resonant modes of the UHQ-MKR still exhibited high stability, with the linewidth fluctuation standard deviation slightly increasing to 4.33 fm (Fig. 4b). This indicates that although the UHQ-MKR relies entirely on the mechanical structure of the knot for coupling and fixation, it remains highly immune to conventional environmental influences. During a long-term 96 h test, due to temperature change in the laboratory environment and the release of residual stress inside the microfiber, the Q-factor of the UHQ-MKR exhibited relatively larger fluctuations, with resonant mode linewidth jitter reaching 22.93 fm. However, overall, the Q-factor remained above 10^7^, as shown in Fig. 4c. More data with ambient temperature and humidity as well as package-enhancement strategy can be found in Supplementary Note 5.

The thermal effects inside the UHQ-MKR were also investigated. As shown in Fig. 4d, e, with increasing input optical power, the device exhibited significant thermal bistability. With the intracavity power building up to a level sufficient to induce a noticeable thermo-optic effect, the refractive index of the cavity material gets changed, introducing a feedback loop that leads to a substantial resonance tilt, ultimately resulting in bistability^45^. In Fig. 4d, the direction of the blue-detuning scan is opposite to the mode shift caused by the thermal effect, leading to a narrowed transmission spectrum. Conversely, in Fig. 4e, the red-detuning scan causes thermal broadening of the resonance mode, resulting in a distinct triangular transmission spectrum, with the thermal shift becoming more pronounced as power increases. Given the low thermal conductivity of air, high-thermal-conductivity substrates and thermoelectric cooler are supposed to be introduced in the next step to ensure the thermal stability of the device.

**Fig. 5:**
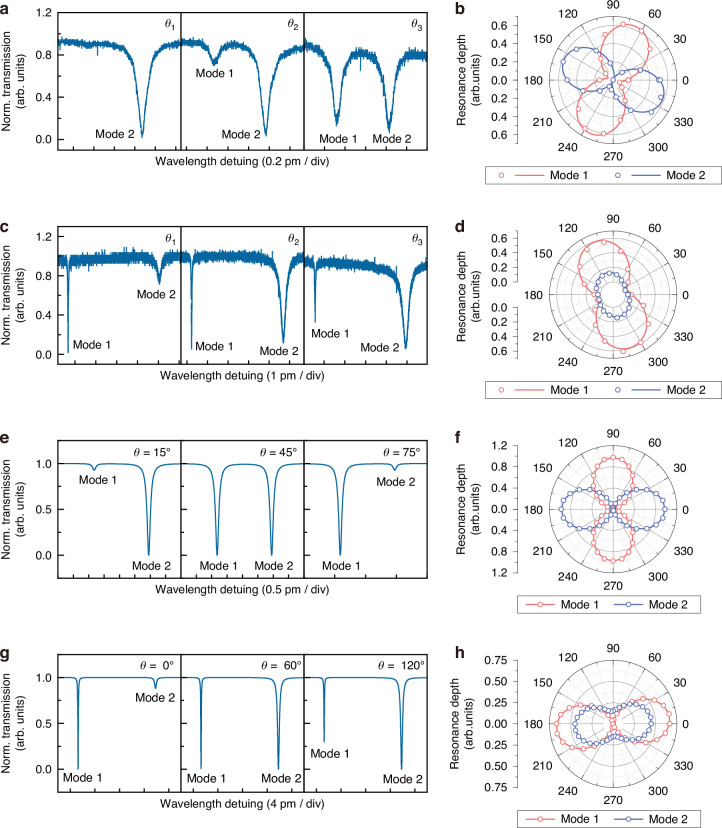
Polarization-dependent transmission of UHQ-MKR. **a** Experimental transmission spectrum for two modes with similar Q-factor in sample (**a**, **b**) the corresponding resonance depth distribution versus polarization angle of incident light for both modes, data fitted by sine function. **c** Experimental transmission spectrum for two modes with significantly different Q-factor in sample (**a**, **d**) the corresponding resonance depth distribution versus polarization angle of incident light for both modes, data fitted by sine function. **e** Simulated transmission spectrum of UHQ-MKR with varied input polarization, where the split modes share similar Q-factor, and (**f**) the corresponding simulated resonance depth distribution versus input polarization angle for both modes. **g** Simulated transmission spectrum of UHQ-MKR with varied input polarization, where the split modes have different Q-factors, and **h** the corresponding simulated resonance depth distribution versus input polarization angle for both modes

Next, being a twisting coupler structure, the knot region should predictably introduce polarization dependent transmission to the MKR. To figure out the polarization features for MKR with UHQ, we replaced the PC in Fig. S1c with linear polarizer and *λ*/2 waveplate for quantitative examination. The differences in the twist states of the knot regions in various samples result in two main distinct polarization dependencies. As shown in Fig. 5a, the transmission spectrum of sample A at different angles indicate that the two resonant modes have similar Q-factors around 1.7 × 10^7^, with periodical alternating changes in the resonance depths. Figure 5b further investigates the relationship between the incident light polarization angle distribution and the resonance depths of the two modes for Sample A. It can be observed that the resonance depths of the two modes exhibit an orthogonal distribution. Another situation is shown in Fig. 5c, where the transmission spectra of sample B at different angles reveal a significant difference in the Q-factors of the two modes, one exceeds 1.2 × 10^7^ (Mode 1) while another is only around 4.6 × 10^6^ (Mode 2). Despite the resonance depths of the two modes alternate at certain angles in sample B, in general they change with the same trend when the polarization angle of the incident light rotates (Fig. 5d). We also investigated the relationship between the mode spacing of the two resonant modes and the polarization angle, and the results show that the mode spacing remains unchanged, independent of the polarization angle of the incident light. Besides, Study of the relationship between the Q-factor and the polarization of input light in UHQ-MKR shows that the Q-factors do not change with the polarization angle of the incident light either (see Supplementary Note 6).

Here an amended model is proposed to add birefringence into the MKR spectrum transmission matrices for numerical explanation to the polarization-sensitive responses of UHQ-MKR, as introduced in Supplementary Note 7. In this model, the key parameters that determine the polarization-dependent transmission of MKR are the birefringence $B$, the coupling coefficients in circularly modified coupled mode equations ${\kappa }_{a}=\left({\kappa }_{x}+{\kappa }_{y}\right)/2$ and ${\kappa }_{b}=\left({\kappa }_{x}-{\kappa }_{y}\right)/2$, where ${\kappa }_{x}$ and ${\kappa }_{y}$ are the deviated coupling coefficients for the two eigenstates of polarization respectively.

First, we assume the condition where the birefringence is relatively small and the coupling coefficients for both eigenstates of polarization are still degenerate, i.e. the *κ*_*b*_ =0 μm^-1^. Here the birefringence *B* is set to be 3 × 10^−5^ and *κ*_*a*_ = 7.95 × 10^−3^ μm^−1^. The over-all length of cavity *L*_k _+ *L*_r_ = 7.5 mm with knot coupling area length of *L*_k_ = 400 μm. Figure 5e shows the simulated resonance spectrum of an UHQ-MKR sample, where the split modes have identical Q-factors of 1.2 × 10^7^, and the incident light polarization angles are 15°, 45°, and 75°, respectively. As seen in Fig. 5f, the resonance depths of the two modes exhibit an orthogonal distribution with respect to the input polarization angles, demonstrating that they belong to different polarization eigenstates. Meanwhile, both the Q-factors of two modes show subtle variations with respect to the polarization angle of the incident light (Fig. S7c). Such condition coordinates with the experimental results of sample A.

Next, the condition where the birefringence is relatively larger and the coupling coefficients for both eigenstates of polarization keenly diverge is investigated. Here we set the *κ*_*b*_ = 4 × 10^-3^ μm^−1^) and *B* = 5 × 10^−4^, while other parameters remain unchanged. Figure 5g shows the resonance spectrum under such circumstance, where the split modes have significantly different Q-factors, and the incident light polarization angles are 0°, 60°, and 120°, respectively. Mode 1 exhibits Q of 1.7 × 10^7^ while Mode 2 only reaches 4.5 × 10^6^. As seen in Fig. 5h, the resonance depths of the two modes change in the same direction with the input polarization angles, only alternate at certain small angle range. Though diverging, the Q-factors of both modes are still independent of the polarization angle of the incident light (Fig. S7d). The theoretical results are consistent with the experimental findings in sample B.

### Single-frequency fiber laser based on UHQ-MKR

To highlight the value of ultra-high Q-factor in MKR, we integrated an UHQ-MKR device with Q exceeding 1.3 × 10^7^ solely into an all-fiber laser system to boost narrow-linewidth single-frequency laser output, which are unimaginable for traditional microfiber resonators with Q of only 10^4^.

Single-frequency fiber lasers have garnered widespread research interest due to their advantages of narrow linewidth, excellent monochromaticity, and strong anti-interference capability^46^. To achieve stable single-frequency fiber laser output, it is necessary to suppress multi-longitudinal-mode oscillations in the oscillator, ensuring only one longitudinal mode operates within the gain bandwidth. Here, we utilize only one UHQ-MKR device as a mode-selecting device to directly achieve narrow-linewidth single-frequency laser output.

**Fig. 6:**
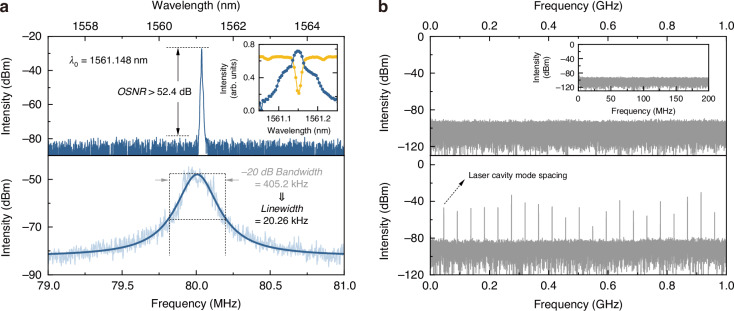
Single-frequency operation of UHQ-MKR based fiber laser. **a** (top panel) Optical spectrum of single-frequency laser output, inset shows the relative spectral position of the laser (blue curve) and MKR resonant mode (yellow curve). (lower panel) Linewidth measurement result of the single frequency laser with Lorentz fitting. **b** (top panel) RF spectrum of single-frequency laser output within 1 GHz range, inset shows scanning of the same output within 200 MHz range. (lower panel) RF spectrum of free-running fiber laser without UHQ-MKR in 1 GHz range

Based on the experimental all-fiber laser scheme shown in Fig. S10a, by tuning the intracavity polarization state to efficiently excite the high-Q resonant mode of the UHQ-MKR, the laser generates single-frequency output with a high optical signal-to-noise ratio (OSNR) at the wavelength corresponding to this resonant mode (top panel of Fig. 6a). At a pump driven current of 800 mA, the laser’s central wavelength is 1561.148 nm, with an OSNR exceeding 52.4 dB. The inset shows the relative positions of the laser spectrum and the transmission spectrum of the UHQ-MKR sample, confirming that the emission wavelength aligns well with the device’s resonant mode. Laser output generating at the resonances of UHQ-MKR might be counterintuitive since the resonant frequency seems experiencing larger roundtrip loss in the laser cavity. This can be explained by the Fermi’s golden rule^47,48^, with detailed explanation in Supplementary Note 9.

Thanks to the UHQ-MKR’s Q-factor exceeding 10^7^, its resonant mode linewidth is <20 MHz. Considering that the longitudinal mode spacing of fiber lasers typically ranges from tens of MHz, this narrow linewidth is highly advantageous for mode selection, effectively ensuring single-longitudinal-mode oscillation in the fiber laser. Using the delayed self-heterodyne method (see Supplementary Note 9 for details), we characterized the linewidth of the obtained single-frequency laser (lower panel of Fig. 6a). The measured beating note can be well-fitted with a Lorentzian lineshape, yielding a 20 dB linewidth of ~405.2 kHz. Accounting for the inevitable power attenuation and 1/*f* noise introduced by the kilometer-scale ultra-long delay fiber^49^, the actual linewidth of the laser output is estimated to be about 20.26 kHz. Further optimization of the fiber laser cavity configuration may enable even narrower linewidths in the future.

The single-longitudinal-mode operation of the UHQ-MKR-based laser also gets proved by using the self-homodyne method. In the top panel of Fig. 6b, the radio-frequency (RF) spectrum in 0$\sim$1 GHz range is illustrated, with 0$\sim$200 MHz range in the inset. No obvious beating signal is observed in both situations, indicating stable single-longitudinal-mode operation and exceptional mode-selecting performance of the UHQ-MKR. To verify this performance, the device is replaced by a section of SMF with similar length, and the corresponding RF spectrum of the laser output is shown lower panel of Fig. 6b, which exhibits dense beating notes representing the laser cavity mode spacing and its harmonics, demonstrating obvious multi-longitudinal-mode oscillation.

Furthermore, owing to the narrow linewidth of the generated single-frequency laser and the ultra-high Q-factor of the UHQ-MKR, the threshold for nonlinear parametric processes in the device can be significantly reduced to a level achievable by this laser setup, enabling four-wave-mixing parametric oscillation^50^, as demonstrated in the Supplementary Note 9.

## Discussion

In summary, we have developed a precision fabrication workflow that elevates the Q-factor of MKRs to 10^7^ level, nearly three orders of magnitude higher than conventional values and representing the highest Q ever reported for microfiber-based devices. Through the first systematic investigation of the long-standing Q bottleneck in MKRs, we identified two fundamental limiting factors: microfiber mechanical quality and knot-region coupling control. Specifically, we established a coupling model to determine the optimal knot geometry, and revealed that ambient-sensitive hydrogen-oxygen flame processing directly influences the stress distribution and uniformity of the microfiber. By regulating environmental temperature and humidity during fabrication, we achieved microfibers with improved mechanical stability and more uniform stress profiles, enabling reproducible UHQ performance. The fabricated UHQ-MKRs exhibit clear thermal bistability and polarization-dependent transmission, confirming their high optical finesse and stability. A demonstrative application of the UHQ-MKR in the fiber laser scheme was also performed to highlight the value of the high Q-factor, successfully enabling single-frequency laser operation.

Although it should be acknowledged that the current fabrication paradigm remains at the laboratory demonstration stage, it still holds promise for extension into larger-scale production. The proposed steps are primarily part of the one-time determination of optimal fabrication parameters, rather than individualized tuning for each sample. Once the optimal microfiber dimensions, coupling length, and ring size are established, devices fabricated under controlled temperature-humidity conditions can achieve high consistently, indicating good reproducibility.

Rather than aiming to replace classical WGM platforms which still hold the highest absolute Q-factors, this work advances a complementary resonator architecture that combines ultra-high Q with mechanical robustness, monolithic fiber compatibility, and intrinsic flexibility. These unique traits position MKRs for application scenarios less accessible to bulky or chip-based microcavities, such as wearable sensing, underwater acoustics, and mechanically reconfigurable photonic systems. Looking ahead, the combination of our environmental control strategy, coupling model, and scalable fabrication concepts provides a promising pathway toward higher Q limit, while enabling new device concepts in cavity-enhanced wearable sensing, flexible optomechanics, and even quantum state manipulations.

## Materials and methods

### Fabrication workflow of MKR

The fundamental fabrication flow of MKR involves 3 main steps: microfiber tapering, knotting and coupling tuning, summarized as “TKT” method, as illustrated in Fig. S1a.

#### Step1: Tapering of microfiber

Uniformly smooth surfaces, low loss, and consistent shape are critical for achieving UHQ MKR. We employed the flame brushing method for microfiber fabrication, utilizing high-purity hydrogen as the heat source, the whole system is shown in Fig. S1b. The mechanical pulling system includes a hydrogen generator, a digital mass flow controller, a fixed hydrogen flame nozzle, and two one-dimensional high-precision motorized positioning stage, on which two fiber clamps are mounted respectively. The MFC is used to adjust the hydrogen flow rate to maintain consistent flame conditions for each process. The positioning stage is controlled by a LabVIEW program, allowing for dynamic control of the pulling speed. The entire system is located in a cleanroom at 1000, equipped with temperature and humidity control devices.

The standard communication single-mode fiber is mounted on the fiber clamps and is pulled in opposite directions at a steady speed until the diameter of the waist region is reduced to ~3 μm. In our experiment, the flame scanning speed was set to 0.02 mm s^−1^, the single-side tapering speed was controlled at 0.02 mm s^−1^, and the total heating time was ~27 min, during which constant tension was maintained. Simultaneously, the loss of the microfiber during the pulling process is monitored in real-time using an amplified spontaneous emission source and an optical spectrum analyzer (AQ6370C, Yokogawa).

#### Step2: Knotting of microfiber

First, the microfiber should be carefully detached from the motorized positioning stage. Owing to the microfiber’s strong mechanical properties, it can be manually lifted without risk of breakage. Next, we coordinate both hands to tie a large loop, gradually reducing the size of the loop while holding the knot area. When the knot region approaches the microfiber area, the entire structure is placed back onto the motorized positioning stage. Finally, with the assistance of the motorized positioning stage, we slowly reduce the size of the loop while monitoring the position of the coupling region until it is positioned at the waist of the microfiber, which makes the prerequisite for subsequent fine adjustments.

#### Step3: Tuning the coupling of MKR

When the knot region is positioned at the waist of the microfiber, the evanescent field of the microfiber facilitates the formation of coupling in the knot region, favoring resonance within the cavity structure. Again we use the motorized positioning stage for precise coupling adjustments. The pulling speed is real-time manipulated to fine-tune the shape of knot and ring area while simultaneously observing via a microscope. When the coupling length in the knot region matches the size of the ring area, optimal coupling is achieved.

### Measurement and Monitoring of Q-factor

The setup of detuning-scan system is illustrated in Fig. S1c. The output of a wavelength tunable laser (TSL-710, Santec) with linewidth <100 kHz passes through a variable optical attenuator and a polarization controller. A portion is split at the coupler for power monitoring before entering the MKR sample. The transmitted light is detected by a photodetector and measured with a high-speed oscilloscope (DSOX6004A, Keysight), while another portion is sent to optical spectrum analyzer (AQ6370C, Yokogawa) for coarse monitoring. In the experiment, the tunable laser and the oscilloscope are triggered synchronously by a triangular wave generated from an arbitrary signal generator for fine wavelength tuning. Under such trigger mode the scanning resolution can be further promoted to <10 MHz. The time coordinate t of the data collected by the oscilloscope can be converted to wavelength coordinates using the following formula $\lambda ={\lambda }_{0}+a\cdot b\cdot t$, where *λ*_0_ is the reference central wavelength of the tunable laser, *a* is the piezoelectric coefficient, and *b* is the slope of the triangular wave. In this experiment, *a* = 40 pm V^−1^, and *b* = 300 V s^−1^.

## Supplementary information

Supplementary Information for Microfiber knot resonator with 10<sup>7</sup> Q-factor record

## Data Availability

Data underlying the results presented in this paper may be obtained from the authors upon reasonable request.
