# Supplementary material for: Microfiber knot resonator with 107 Q-factor record
Source: Light Sci Appl. 2026 Mar 6;15:155. doi: 10.1038/s41377-025-02124-1 (PMC12966411; doi:10.1038/s41377-025-02124-1)
Supplement: Supplementary file 1 — Supplementary Information for Microfiber knot resonator with 107 Q-factor record [file 41377_2025_2124_MOESM1_ESM.docx]

Supplementary Information for:

**Microfiber knot resonator with 10^7^ Q-factor record**

Xinxin Zhou^1, #^, Zixuan Ding^1, #^, and Fei Xu^1,2, *^

^1^ National Laboratory of Solid-State Microstructures and College of Engineering and Applied Sciences, Nanjing University, Nanjing, 210023, China

^2^ Shenzhen Research Institute of Nanjing University, Shenzhen, 518000, China

^#^ These authors contributed equally to this work.

[*feixu@nju.edu.cn](mailto:*feixu@nju.edu.cn)

**Supplementary Note 1: Fabrication and Characterization of MKR**


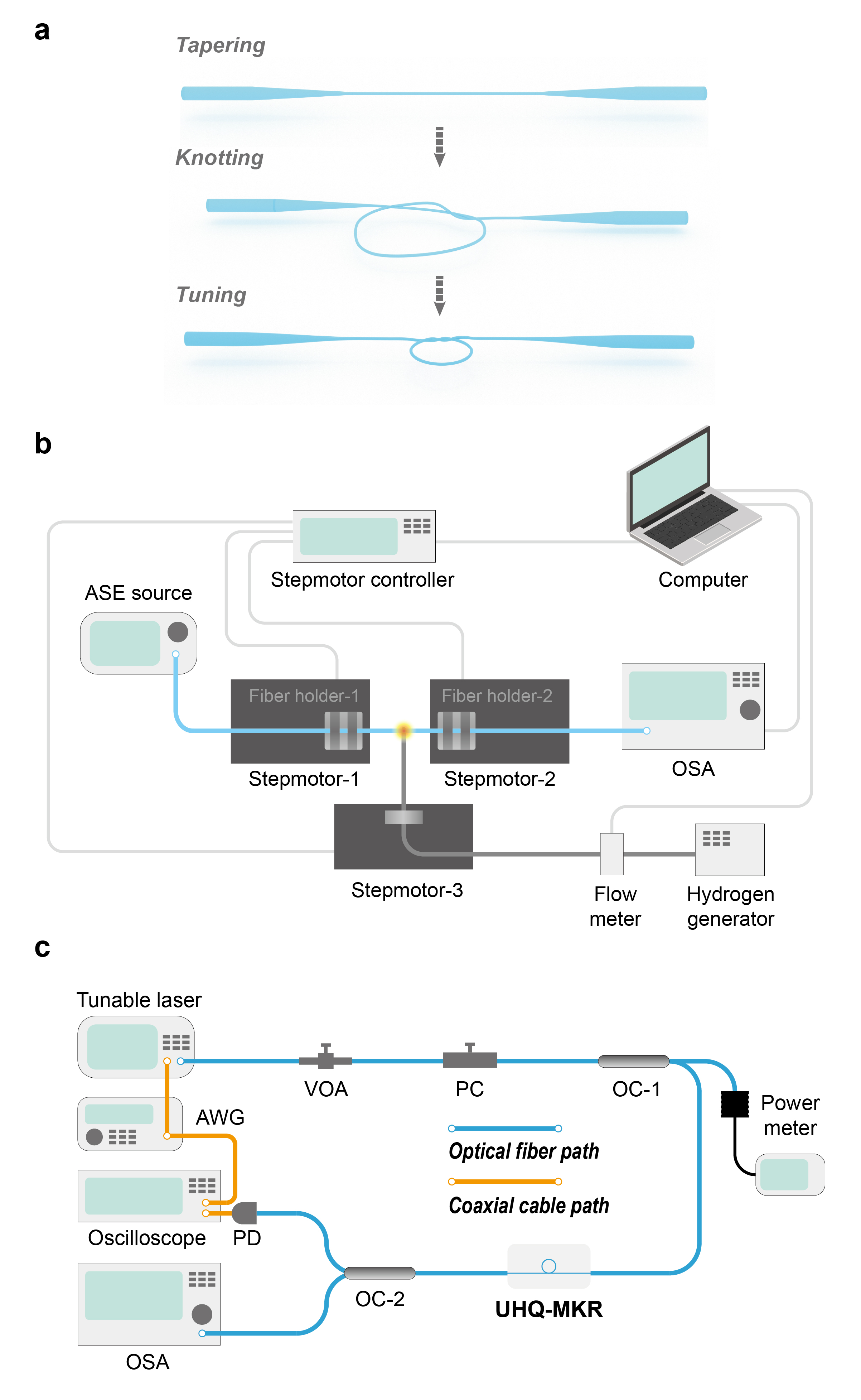


**Figure S1. a** Fabrication flow of microfiber knot resonator. **b** System scheme of flame-brushing tapering technique. **c** Characterization system of ultra-high-Q MKR samples. VOA, variable optical attenuator; PC, polarization controller; OC, optical coupler; PD, photodetector; OSA, optical spectrum analyzer; AWG, arbitrary waveform generator.

Supplementary Note 2: Ultra-low-loss microfiber

An extremely high intrinsic Q-factor is the prerequisite for achieving an ultra-high loaded Q-factor, and for a microfiber-based resonator, this requires that the microfiber waveguide itself has a very low transmission loss. Since the concept of optical fiber microwires/nanowires was proposed, researchers have been committed to reducing their transmission losses via fiber tapering or other methods [1-4].


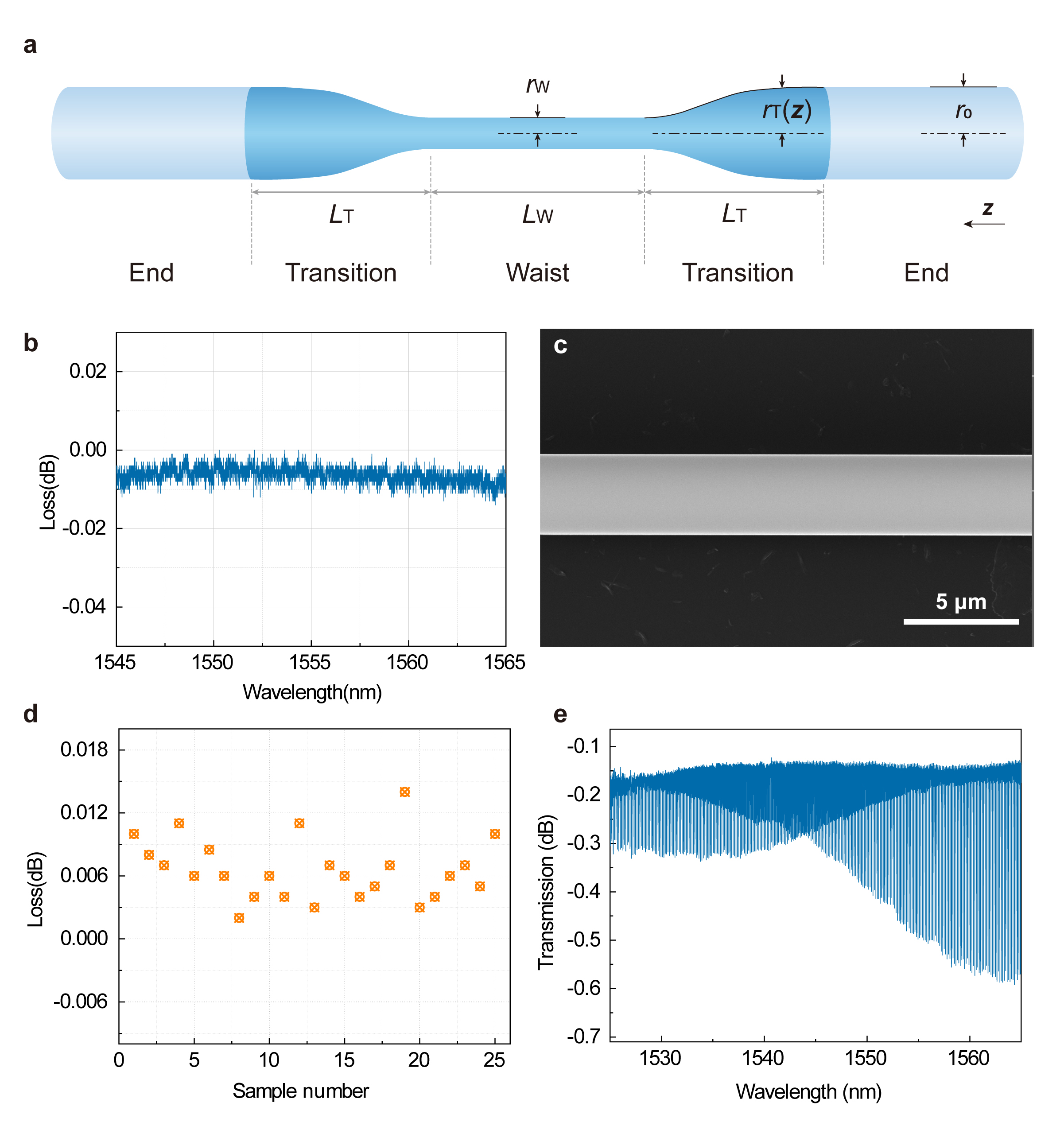


**Figure S2. a** Schematic diagram of microfiber waveguide. **b** Typical transmission spectrum of ultra-low-loss microfiber. **c** Localized SEM photo of ultra-low-loss microfiber. **d** Statistical distribution of transmission loss across multiple batches. **e** Measured non-resonant insertion loss of a typical UHQ-MKR.

The structure of a microfiber drawn from standard commercial optical fiber generally consists of three parts: waist region, transition region, and pigtail portion, as shown in **Fig. S2a**. The keys to loss management involve two aspects: material and geometric shape. As for the material, the appropriate heating and annealing methods should be selected during the preparation process to ensure the material homogeneity of silica during the melting and re-solidification process, avoiding the intrinsic absorption and scattering losses of the material. As for the geometric shape, on the one hand, it is necessary to attain a gentle transition zone of sufficient length so that the standard fiber guided mode is gradually transformed into the microfiber mode to avoid the severe mode leakage loss caused by the sharp narrowing of the waveguide; on the other hand, it is vital to ensure that the diameter of the waist area is uniform and the surface is smooth, otherwise it will cause huge losses due to mode fluctuations and interface scattering. The propagation loss $\alpha$ is related to the propagation constant $k$, the absolute value of the transversal component of the propagation constant $\delta$ and the characteristic length of diameter fluctuations $L_{f}$ by [5]

$$\alpha=\frac{1}{4\delta}\sqrt{\frac{k}{L_{f}}}\exp\left( -\frac{\pi L_{f}\delta^{2}}{k} \right)$$

(S1)

It has been demonstrated that the flame-brushing and modified flame-brushing techniques provide the lowest loss across a wide range of microfiber diameters [6]. Based on the monitoring setup introduced in Supplementary Note 1, the average loss of the microfiber in our experiment is approximately ${10}^{-3}$ dB mm^-1^, reaching the optimal state of existing microfibers. Typical transmission spectrum is illustrated in **Fig. S2b**, and the statistical distribution of transmission loss across multiple microfiber batches is presented in **Fig. S2d**. The SEM photo shown in **Fig. S2c** exhibits perfectly high surface smoothness, implying ultra-low scattering loss.

Owing to the low transmission loss of our fabricated microfiber, the UHQ-MKR also features very low insertion loss. Under typical experimental conditions, the insertion loss of the resonator in the non-resonant spectral region is approximately 0.2 dB, as shown in **Fig. S2e**. This loss primarily arises from modal mismatch introduced during the tapering process and minor scattering at the fusion splice points. Thanks to the structural compatibility of the microfiber resonator with standard fiber systems and the all-fiber configuration of our device, no additional coupling platforms or high-precision alignment systems are required. As a result, the overall coupling loss is significantly reduced, enabling low insertion loss while maintaining excellent stability and integration potential.

Multiple studies have demonstrated the limitations of conventional coupling schemes in terms of insertion loss. For example, Rasoloniaina *et al.* analyzed the coupling characteristics of erbium-doped WGM microcavities and reported that the insertion loss could reach as high as 52 dB under critical coupling conditions, indicating extreme sensitivity to coupling parameters and significant energy loss [7]. Similarly, Mei *et al.* developed a WGM microsphere resonator based on cylindrical air cavity coupling, which exhibited an insertion loss of approximately 33 dB [8]. In a recent review of various in-fiber WGM microsphere coupling configurations, Yang *et al.* pointed out that conventional coupling techniques (such as D-shaped fibers and angle-polished end faces) are prone to structural misalignment, often resulting in insertion losses exceeding 18 dB [9].

In contrast, the knot-type microfiber resonator adopts an all-fiber connection architecture, eliminating the need for any external alignment optics or free-space coupling structures. This enables a stable and low insertion loss as low as 0.2 dB in practical systems. Such low-loss characteristics not only improve overall system energy efficiency but also offer significant advantages for real-world applications.

To enhance the Q-factor to an even higher level, we summarize some key strategies as follows:

***Further optimization of the tapering process to reduce transmission loss:*** According to the experimental study by Hoffman *et al.*, precise control of thermal field stability and mechanical tension during the tapering process can significantly reduce the likelihood of non-adiabatic excitation of higher-order modes and enhance the symmetry of fundamental mode propagation [10]. This leads to a notable reduction in transmission loss (down to 2.6×10^−5^ dB mm^-1^), and thereby improves the overall Q-factor of the resonator. In our work, the tapering was performed using a flame-scanning method. To ensure thermal field stability, we maintained a controlled temperature and humidity environment during the process. In future work, we plan to replace the flame-scanning setup with a fixed oxyhydrogen flame or an electric heater, which could further enhance the stability of the flame, maintain a uniform thermal field, and reduce the impact of airflow fluctuations.

***Enhancing adiabaticity during the tapering process to suppress mode asymmetry:*** Zhang *et al.* have pointed out that the taper angle and the length of the transition region must strictly satisfy adiabatic conditions in order to suppress coupling from the fundamental mode to higher-order or radiation modes [11]. In practice, this can be achieved through controlled “flame-brushing” techniques, enabling taper angles to be maintained within a range of less than 1°. Combined with a real-time diameter monitoring system with feedback control, this approach helps preserve mode integrity throughout the tapering process.

***Improving surface quality and contamination control:*** Hoffman *et al.* found that even minimal surface contamination or residual coatings can excite asymmetric modes, increase loss, and significantly reduce the Q-factor [10]. By adopting cleanroom techniques—such as using high-purity gases (filtered to 0.003 µm) and optimizing the pre-tapering cleaning procedures—the surface condition of the microfiber can be effectively improved, thereby suppressing absorption and scattering losses.

Supplementary Note 3: Mechanical properties and bending loss of microfiber

Despite minimal size, microfibers exhibit an extraordinarily high strength. The fracture strength (defined as the maximum stress a microfiber can withstand before fracture occurs) of samples manufactured by the flame-brushing technique got measured via simple elongation experiments and the results are shown in **Fig. S3a**. The results show fracture strength around 10 GPa level, which is consistent with previous results of silica nano/microwires [11, 12].


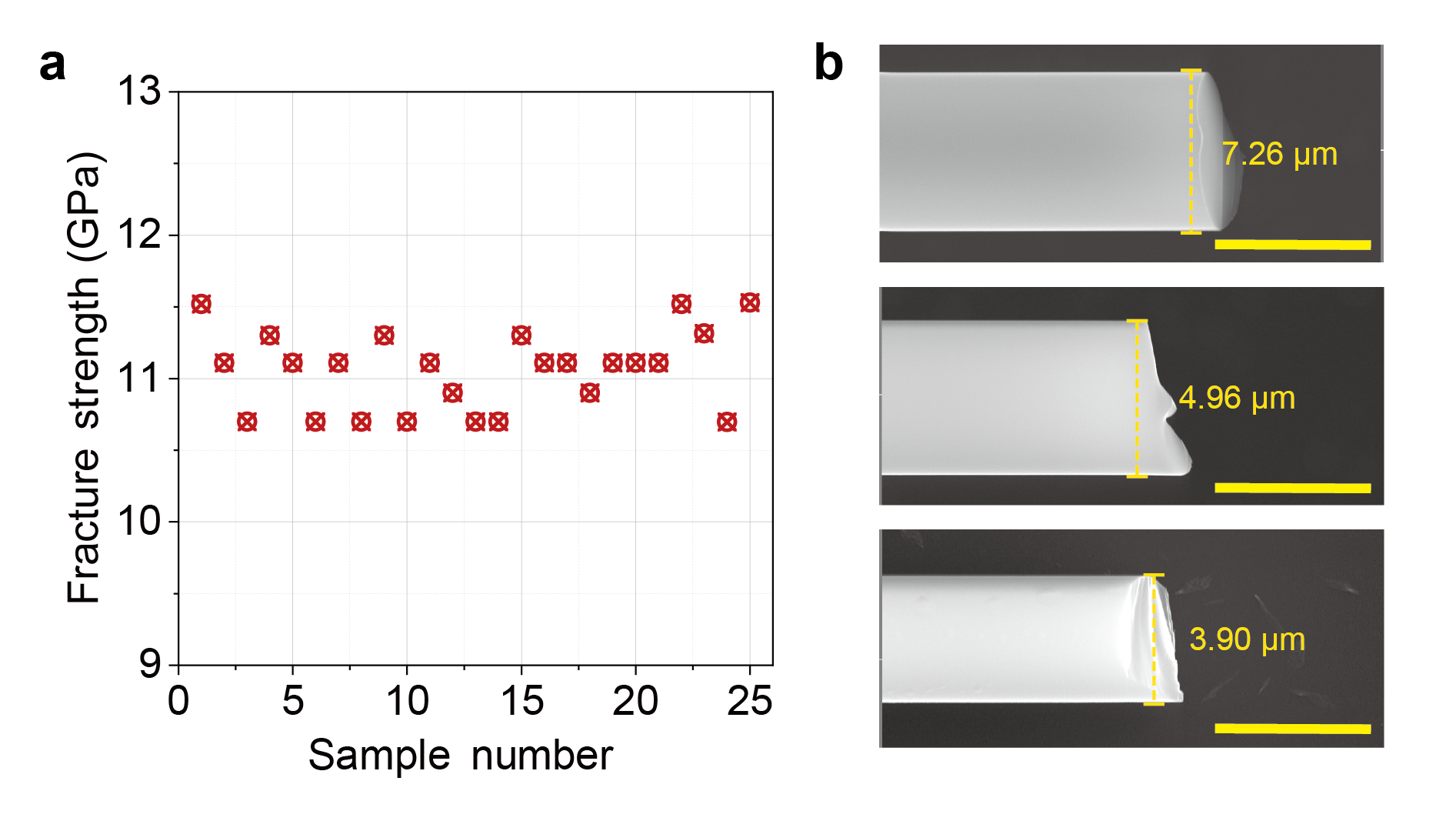


**Figure S3. a** Fracture strength of microfiber waveguide measured with elongation experiments. **b** SEM photos for breaking points of microfibers prepared under different environments, scale bars represent 5 μm.

The SEM photos of breaking points of microfibers prepared under different environmental conditions were also taken to characterize the fracture process. As can be seen in **Fig. S3b**, environmental parameters affect the breaking position evidently by forming varied inner stress distribution in the microfiber. Such distribution would thereby influence the Q-factor of MKR though determination of knot coupling area’s geometrical structure.

To deal with the potential risk of fiber breakage during the sample fabrication process, we optimized the working flow from both the experimental design and operational procedure perspectives. We strictly controlled temperature, humidity, and other critical parameters throughout the experiment. During the microfiber fabrication process, we precisely adjusted the pulling rate and heating temperature to ensure good mechanical uniformity and strength, thereby preventing the formation of local micro-cracks and providing a reliable foundation for subsequent knotting operations. Moreover, by finely tuning the ambient temperature and humidity, we ensured a uniform thermal field distribution, avoiding uneven internal stress caused by environmental fluctuations, which further contributed to a more uniform stress distribution during both the tapering and knotting processes. In addition, during the knotting procedure, we employed a micro-positioning stage to manipulate the microfiber slowly and precisely, avoiding any abrupt stretching or sudden stress application. Through these measures, we successfully reduced the risk of fiber breakage and ensured the mechanical robustness and stability of the microfiber during knotting, thereby enabling the final MKR devices to achieve higher Q-factors and improved operational stability.

Another concern regarding the mechanical structure of MKR is the bending loss. To evaluate the potential impact of bending on the Q-factor in the MKR, we conducted the following simulation analysis and theoretical investigation on bending loss:

First, as shown in **Fig. 3b** of the main text, the free spectral range (FSR) of the UHQ-MKR is typically less than 25 GHz. According to the relation $FSR=c/(n_{\mathrm{eff}}\cdot2\pi R)$, where $c$ is the speed of light in vacuum and $n_{\mathrm{eff}}$​ is the effective refractive index, we can estimate that the typical bending radius $R$ corresponding to this type of structure is greater than 500 μm.

To investigate whether significant bending loss occurs in the microfiber under this configuration, we conducted simulation analyses using COMSOL Multiphysics, taking a 3 μm-diameter air-clad microfiber as an example. It is important to note that microfibers inherently exhibit strong evanescent field characteristics, meaning that the guided mode energy is not fully confined within the core, but partially leaks into the cladding. Therefore, in our loss evaluation, we focused on the additional radiation loss introduced by bending.

First, we transformed the bent fiber into an equivalent straight waveguide and incorporated the stress-optic effect to construct the effective refractive index distribution under bending [13, 14]. Based on this, we established an accurate bending loss model in COMSOL. By numerically calculating the mode field distribution of the bent microfiber, we then computed the effective refractive index for different bending radii. Specifically, the bending of the fiber induces localized changes in the refractive index profile, leading to partial leakage of the mode energy. The corresponding leakage energy was calculated to evaluate the bending-induced loss.

**Fig. S4a** shows the mode field distribution of a straight microfiber. Although an evanescent field is present, the mode remains largely confined near the fiber core, and no additional radiation loss due to bending is observed. However, when the bending radius is reduced to 15 μm, the mode field expands significantly and leaks into the surrounding air cladding, indicating the presence of pronounced bend-induced radiation, as shown in **Fig. S4b**.


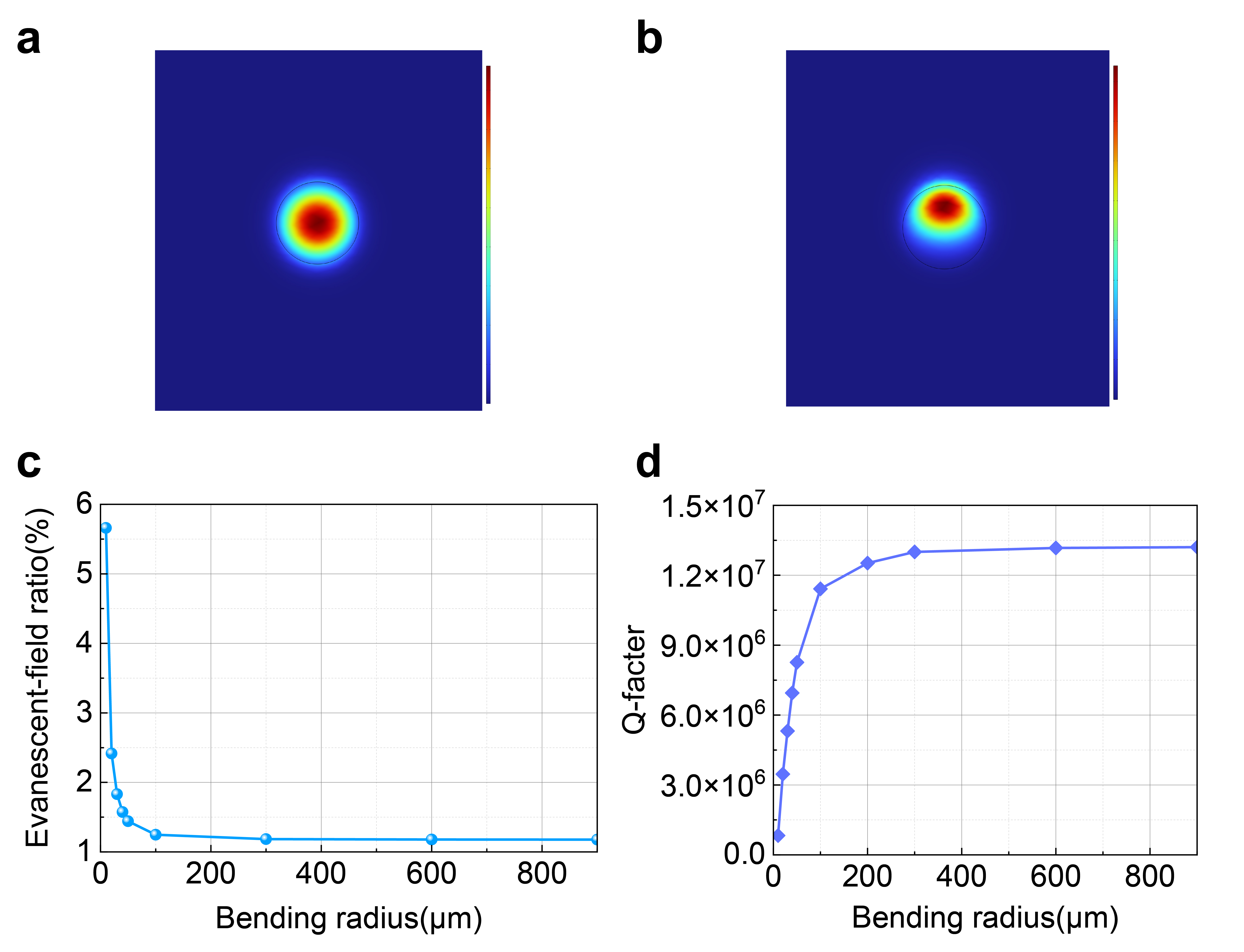


**Figure S4. Radiation loss analysis of the microfiber under different bending radii.** **a** Mode field distribution without bending. **b** Mode field distribution at a bending radius of 15 μm. **c** Evanescent energy ratio vs. bending radius for 3 μm microfiber. **d** Theoretical Q-factor vs. bending radius for 3 μm microfiber.

To quantitatively evaluate this loss, we further calculated the evanescent field energy ratio at different bending radii, which is defined as the proportion of modal energy located outside the fiber core region relative to the total energy. A higher ratio indicates greater mode leakage into the cladding, i.e., more significant radiation loss. As shown in **Fig. S4c**, this ratio increases rapidly when the bending radius falls below approximately 100 μm, suggesting a substantial rise in bending loss. In contrast, when the bending radius exceeds 500 μm, the additional radiation loss becomes negligible and has bare impact on the Q-factor, as shown in **Fig. S4d**.

Therefore, we conclude that in the actual working structure, the additional radiation loss caused by bending is negligible and has only a minimal impact on the Q-factor. Meanwhile, in the literature *“Microfiber Resonator in Polymer Matrix,”* the authors adopted an alternative approach to evaluate bending loss by indirectly inferring its effect on the Q-factor through variations in the FSR observed in the resonator’s output spectrum [15]. Their results similarly indicate that a ring region diameter greater than 500 μm does not introduce noticeable bending loss. Consequently, the Q-factor degradation observed in our study is more likely attributed to material absorption, surface scattering due to roughness, and coupling losses, rather than to bending itself.

Supplementary Note 4: Theoretical analysis of MKR coupling tuning

Considering microfiber waveguide (refractive index $n_{1}$) with air-cladding (refractive index $n_{0}$), the electromagnetic fields of the fundamental mode in microfiber can be expressed as:

$$0\leq r\leq a$$

$$E_{x}=AJ_{0}\left( \frac{u}{a}r \right)\cos\Psi,$$

$$E_{y}=-AJ_{0}\left( \frac{u}{a}r \right)\sin\Psi,$$

$$E_{z}=i\frac{u}{\beta a}AJ_{1}\left( \frac{u}{a}r \right)\cos\left( \theta+\Psi\right),$$

$$H_{x}=\frac{\omega\varepsilon_{0}n_{1}^{2}}{\beta}AJ_{0}\left( \frac{u}{a}r \right)\sin\Psi,$$

$$H_{y}=\frac{\omega\varepsilon_{0}n_{1}^{2}}{\beta}AJ_{0}\left( \frac{u}{a}r \right)\cos\Psi,$$

$$H_{z}=i\frac{u}{\omega\varepsilon_{0}a}AJ_{1}\left( \frac{u}{a}r \right)\sin\left( \theta+\Psi\right)$$

$$r>a$$

$$E_{x}=A\frac{J_{0}\left( u \right)}{K_{0}\left( w \right)}K_{0}\left( \frac{u}{a}r \right)\cos\Psi,$$

$$E_{y}=-A\frac{J_{0}\left( u \right)}{K_{0}\left( w \right)}K_{0}\left( \frac{u}{a}r \right)\sin\Psi,$$

$$E_{z}=i\frac{u}{\beta a}A\frac{J_{1}\left( u \right)}{K_{1}\left( w \right)}K_{1}\left( \frac{u}{a}r \right)\cos\left( \theta+\Psi\right),$$

$$H_{x}=\frac{\omega\varepsilon_{0}n_{0}^{2}}{\beta}A\frac{J_{0}\left( u \right)}{K_{0}\left( w \right)}K_{0}\left( \frac{u}{a}r \right)\sin\Psi,$$

$$H_{y}=\frac{\omega\varepsilon_{0}n_{0}^{2}}{\beta}A\frac{J_{0}\left( u \right)}{K_{0}\left( w \right)}K_{0}\left( \frac{u}{a}r \right)\cos\Psi,$$

$$H_{z}=i\frac{u}{\omega\varepsilon_{0}a}A\frac{J_{1}\left( u \right)}{K_{1}\left( w \right)}K_{1}\left( \frac{u}{a}r \right)\sin\left( \theta+\Psi\right)$$

(S2)

where $r$, $\theta$ and $\Psi$ are the cylindrical coordinates, $a=D_{F}/2$ is the radius of microfiber cross-section, $\omega$ is the angular frequency, $\varepsilon_{0}$ is the vacuum permittivity, $J_{m}$ represents the *m*th-order Bessel functions, $K_{m}$ represents the *m*th-order modified Bessel functions of the second kind, $u=a\sqrt{k_{0}^{2}n_{1}^{2}-\beta^{2}}$, $v=ak_{0}\sqrt{n_{1}^{2}-n_{0}^{2}}$, and $w=a\sqrt{\beta^{2}-k_{0}^{2}n_{0}^{2}}$ are the normalized transverse wave numbers with vacuum propagation constant $k_{0}$ and guided-wave propagation constant $\beta$. Constant $A$ is related to optical power $P$ by

$$\left| A \right|=\frac{w}{avJ_{1}\left( u \right)}\sqrt{\frac{2P\left( \mu_{0}/\varepsilon_{0} \right)^{1/2}}{\pi n_{1}}}$$

(S3)

Substituting Eqs. (S2) into the mode-coupling coefficient expression:

$$\kappa=\frac{\omega\varepsilon_{0}\iint_{-\infty}^{\infty} \left( N^{2}-N_{2}^{2} \right)\boldsymbol{E}_{1}^{*}\cdot\boldsymbol{E}_{2}dxdy}{\iint_{-\infty}^{\infty} \boldsymbol{u}_{z}\boldsymbol{\cdot}\left( \boldsymbol{E}_{1}^{*}\times\boldsymbol{H}_{1}+\boldsymbol{E}_{1}\times\boldsymbol{H}_{1}^{*} \right)dxdy}$$

(S4)

According to the definition, $\iint_{-\infty}^{\infty} \boldsymbol{u}_{z}\boldsymbol{\cdot}\left( \boldsymbol{E}_{1}^{*}\times\boldsymbol{H}_{1}+\boldsymbol{E}_{1}\times\boldsymbol{H}_{1}^{*} \right)dxdy=4P$. For the remaining integration $S=\iint_{-\infty}^{\infty} \left( N^{2}-N_{2}^{2} \right)\boldsymbol{E}_{1}^{*}\cdot\boldsymbol{E}_{2}dxdy$ on the top, two neighboring microfiber (cylindrical) waveguides model with center separation $D$ is adopted (illustrated in **Fig. S5a**), and $S$ can be approximately derived as [16]

$$S=2\pi a^{2}\left( n_{1}^{2}-n_{0}^{2} \right)\left| A \right|^{2}\frac{J_{0}\left( u \right)}{K_{0}\left( w \right)}\sqrt{\frac{\pi a}{2wD}}\exp\left( -\frac{w}{a}D \right)\times\frac{uJ_{1}\left( u \right)}{{v^{2}K}_{1}\left( w \right)}\times\left[ \frac{J_{0}\left( u \right)}{uJ_{1}\left( u \right)}wK_{1}\left( w \right)I_{1}\left( w \right)+K_{1}\left( w \right)I_{0}\left( w \right) \right]$$

(S5)

Here the $I_{m}$ represents the *m*th-order modified Bessel functions of the first kind. Considering eigenvalue equation of microfiber and relation of modified Bessel functions, the mode-coupling coefficient of the neighboring microfiber waveguide can be obtained:

$$\kappa=\frac{\sqrt{\Delta}}{a}\frac{u^{2}}{v^{3}K_{1}^{2}\left( w \right)}\sqrt{\frac{\pi a}{wD}}\exp\left( -\frac{w}{a}D \right)$$

(S6)

where $\Delta=\left( n_{1}^{2}-n_{0}^{2} \right)/\left( 2n_{1}^{2} \right)$ is the relative refractive-index difference. Considering the adjoined microfiber waveguides as the MKR’s coupling area, $D=2a$, the expression can be simplified as

$$\kappa=\frac{\sqrt{\Delta}}{a}\frac{u^{2}}{v^{3}K_{1}^{2}\left( w \right)}\sqrt{\frac{\pi}{2w}}\exp\left( -2w \right)$$

(S7)

Thereby, the theoretical coupling length can be estimated via $L_{c}=\pi/\left( 2\kappa\right)$. Based on the microfiber-determined coupling coefficient, the Q-factor of MKR in tuning process can be simulated through Eq. (2) in main manuscript.


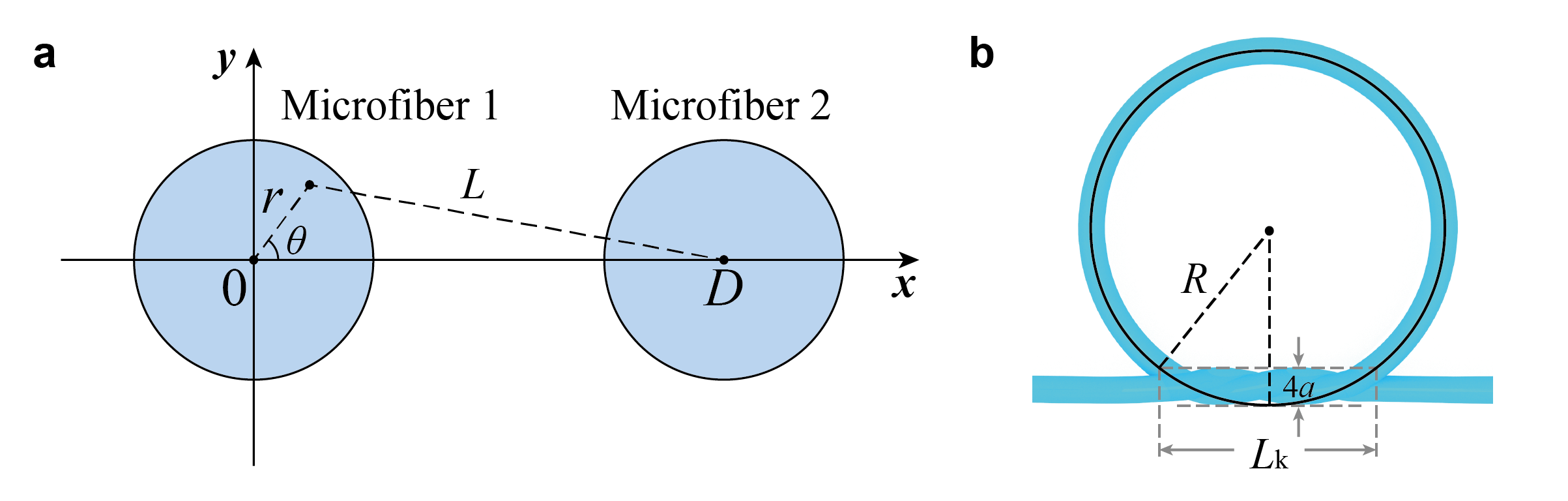


**Figure S5.** Schematic diagrams for **a** neighboring microfiber waveguides coupling model and **b** “standard knot” model.

The geometrical schematic diagram of “standard knot” is shown in **Fig. S5b**, where a perfect circular ring with tight knot area is depicted. The knot effective length $L_{k}$, the microfiber cross-section radius $a$ and the ring section radius $R=D_{R}/2$ meet the Pythagorean relation:

$$\left( \frac{L_{k}}{2} \right)^{2}+\left( R-4a \right)^{2}=R^{2}$$

(S8)

which leads to the expression $L_{k}=2\times2\sqrt{2a\left( R-2a \right)}=4\sqrt{D_{F}\left( R-D_{F} \right)}$.

Supplementary Note 5: Environmental effects on device stability

In this experiment, environmental humidity was carefully controlled. The UHQ-MKR was suspended inside an acrylic enclosure, within which desiccant was placed. Additionally, the laboratory was equipped with a temperature and humidity control system to maintain the relative humidity below 43%. During the experiment, environmental temperature, humidity, and insertion loss were continuously monitored. **Fig. S6** shows the variation trends of the Q-factor recorded over 1 hour, 12 hours, and 96 hours. The influence of humidity on the microfiber surface typically manifests as a reduction in device loss. As shown in **Fig. S6**, no upward trend in loss is observed, suggesting that the Q-factor fluctuations primarily result from temperature variations and residual stress relaxation.


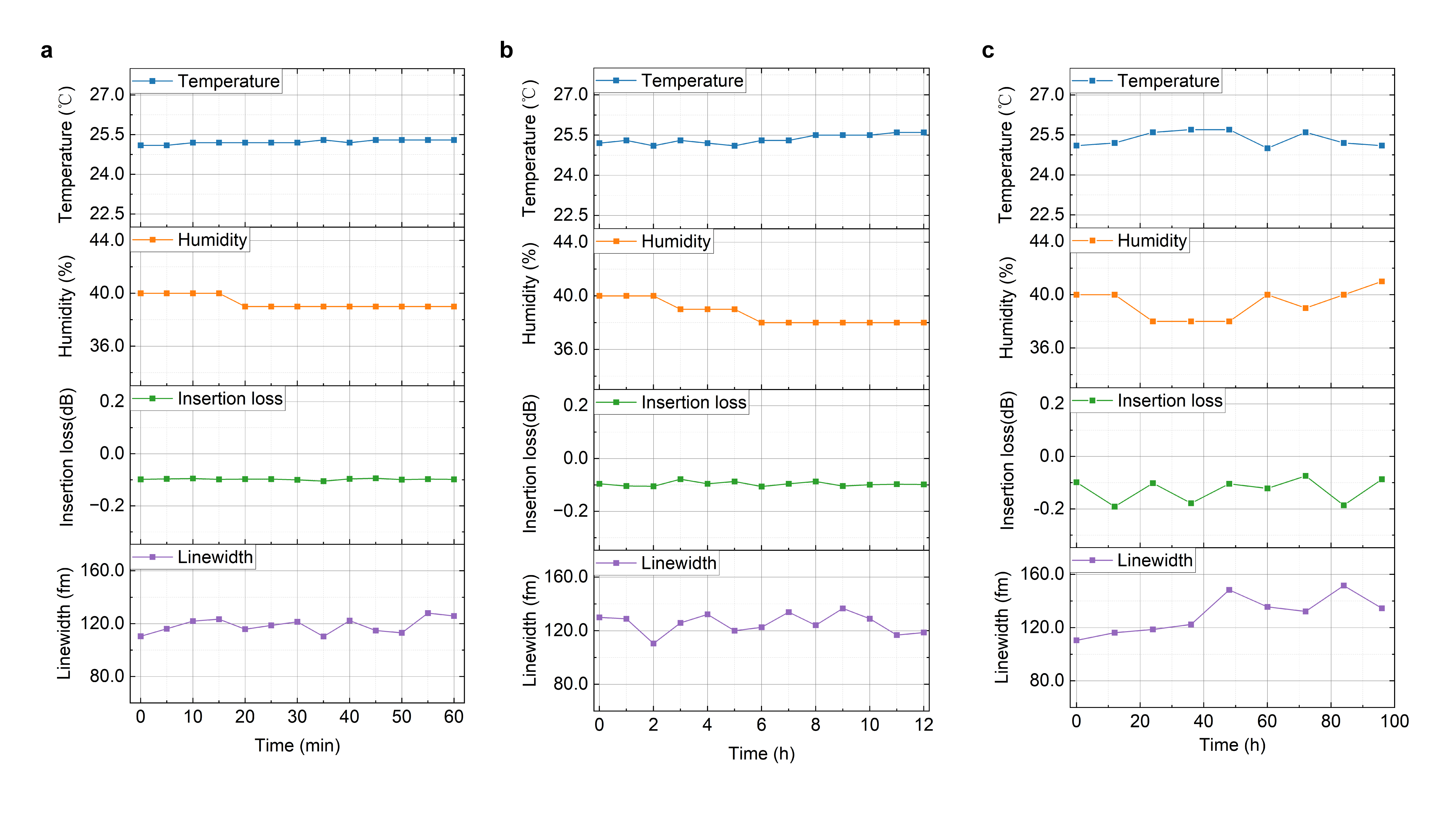


**Figure S6.** Environmental stability test of the UHQ-MKR device. **a** 1 hour, **b** 12 hours, and **c** 96 hours.

The influence of environmental humidity on the microfiber primarily arises from the possible interaction between water molecules in the air and the silica surface, which can lead to the formation of hydroxyl groups. The corresponding chemical reaction is as follows:

$$Si-O-Si+H_{2}O\to Si-OH+HO-Si$$

(S9)

This reaction disrupts the original Si-O-Si bond structure, potentially inducing changes in surface states, increasing hydrophilicity, and consequently enhancing scattering losses, posing a potential threat to the stability of high-Q microfiber devices. However, under ambient conditions of room temperature, atmospheric pressure, and relatively low humidity, this reaction typically does not proceed spontaneously. It generally requires specific conditions (such as elevated temperature, the presence of surface defects or cracks, or laser exposure) to occur significantly, and even then, the process tends to be relatively slow.

In this study, to enhance the structural stability and environmental resilience of the microfiber, we precisely controlled temperature and humidity conditions during the tapering process. This allowed us to achieve a more uniform stress distribution and a smoother fiber surface. The optimized fabrication approach significantly reduced surface defects and hygroscopic sites, thereby effectively extending the onset period of humidity-induced fatigue loss. This improvement is clearly illustrated in **Fig. 2** of the main text.

The influence of relative humidity on microfiber performance is primarily manifested as increased transmission loss, which becomes more pronounced under high-humidity conditions. Previous studies have reported that microfibers can experience losses as high as 10 dB day^-1^ in environments with elevated relative humidity [12]. Such humidity-induced performance degradation has been theoretically explained in the literature through crack growth models [17, 18]. These studies suggest that prolonged exposure to water molecules in the environment can promote the slow propagation of micro-cracks on the fiber surface, leading to stress concentration, enhanced scattering, and gradual deterioration of optical performance, eventually resulting in significant fatigue-type transmission loss. These models provide a physical basis for understanding the effects of humidity on microfiber performance and underscore the importance of environmental control in practical applications of microfiber devices.

However, as shown in the monitoring results in **Fig. S6c**, under a laboratory environment with approximately 40% relative humidity, the microfiber exhibited no noticeable increase in transmission loss over a 96-hour observation period, and the Q-factor remained stable. This experimental result indicates that, in the absence of additional accelerating factors such as external stress or thermal disturbances, moderate humidity conditions do not cause significant loss in our high-quality microfibers over the short term, further confirming their excellent stability under typical ambient conditions.

Nevertheless, from a long-term operational perspective, fatigue-related losses induced by fluctuations in temperature and humidity may still adversely affect the Q-factor stability of UHQ-MKRs. To further enhance the environmental adaptability and operational stability of the device, we plan to implement an encapsulation strategy in future work. Unlike the fully enclosed packaging commonly used for microscale microcavities fabricated from fused silica, which may compromise the high-Q structure, we will design an indirect packaging system specifically tailored for UHQ-MKRs, featuring precise temperature and humidity control. This system will integrate a thermoelectric cooler within the packaging chamber to dynamically regulate temperature and mechanical stress in the coupling region. In addition, high-purity nitrogen gas will be introduced into the chamber to effectively isolate moisture and suppress humidity-induced surface degradation. This packaging strategy is intended to provide stable microenvironmental control without compromising the high-Q structural characteristics, thereby significantly improving the long-term reliability of the device in complex environments.

Supplementary Note 6: Polarization response of UHQ-MKR’s Q-factor


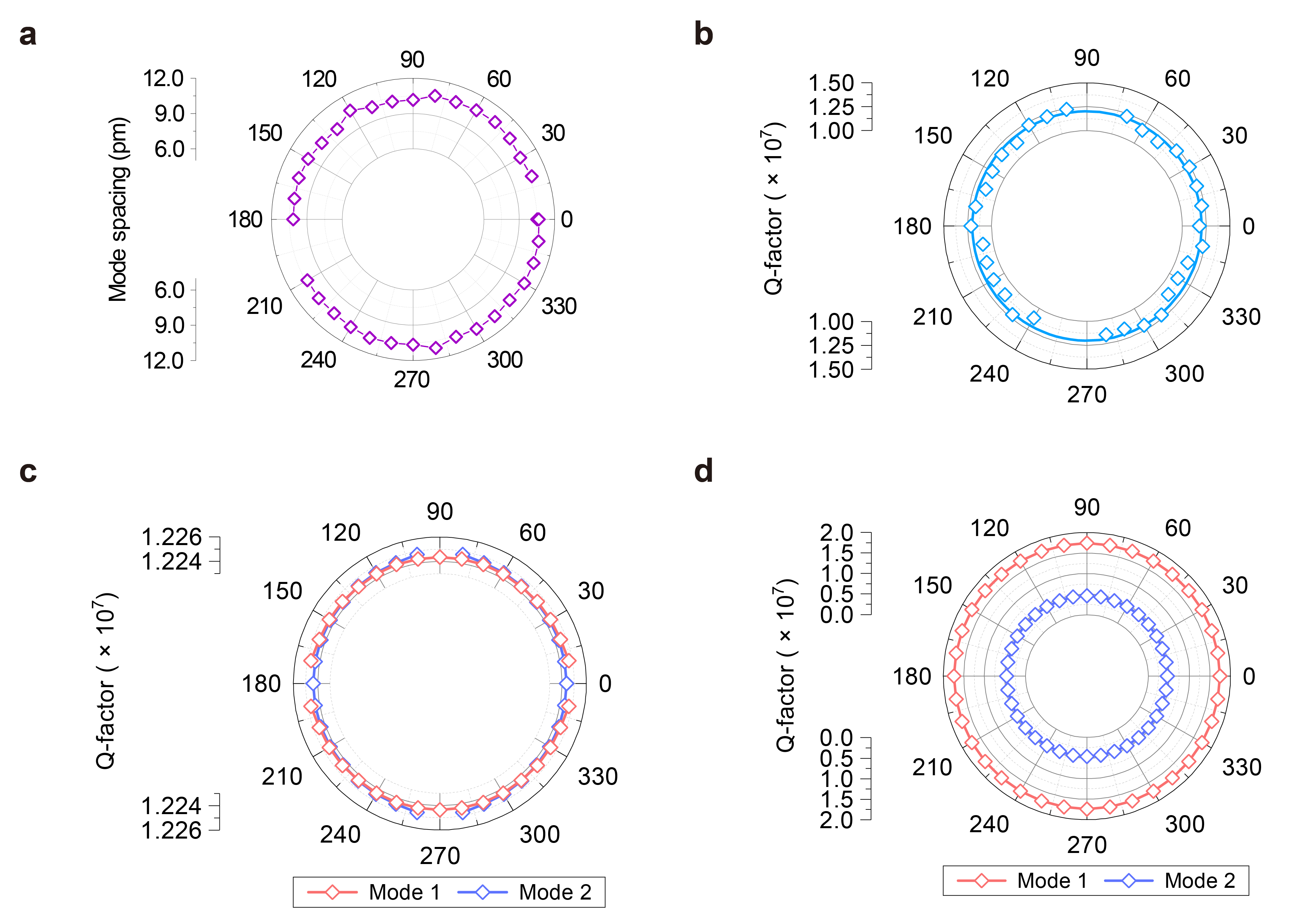


**Figure S7. a** Mode spacing of the UHQ-MKR under different polarization angles. **b** Experimental Q-factor distribution versus polarization angle of incident light for high-Q mode of sample B in the main manuscript. **c** Simulated Q-factor distribution versus input polarization angle for split modes sharing similar Q-factor. **d** Simulated Q-factor distribution versus input polarization angle for split modes with different Q-factors.

Supplementary Note 7: Modified polarization transmission model of MKR

**
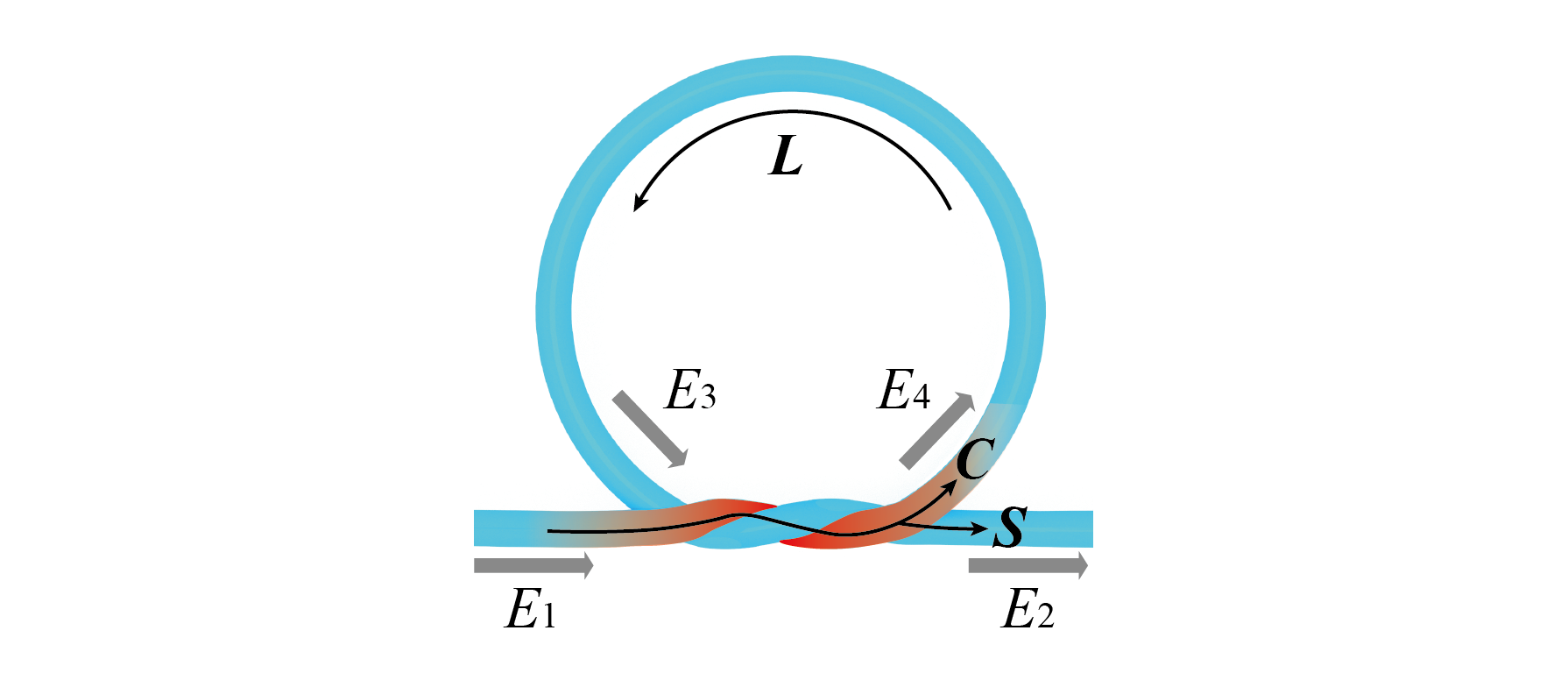
**

**Figure S8.** Polarization transmission of MKR.

The polarization-dependent transmission of MKR can be described through Jones matrix, here we define $E_{j}=\left[ E_{jx}, E_{jy} \right]^{T} ( j=1, 2, 3, 4)$ as the Jones vectors of 4 ports of knot coupling area, and the matrices $S$, $C$, and $L$ represent the self-coupling, cross-coupling, and the ring-section attenuation respectively, as shown in **Fig. S8**. From the model, the electric field vectors satisfy the simple relation:

$$\boldsymbol{E}_{2}=S\boldsymbol{E}_{1}+C\boldsymbol{E}_{3}$$

$$\boldsymbol{E}_{4}=C\boldsymbol{E}_{1}+S\boldsymbol{E}_{3}$$

$$\boldsymbol{E}_{3}=L\boldsymbol{E}_{4}$$

(S10)

Thereby the transmission can be derived as:

$$\boldsymbol{E}_{2}=\left[ S+CLC+CLS{(1-LS)}^{-1}LC \right]\boldsymbol{E}_{1}$$

(S11)

In previous investigation, the birefringence for guided modes in knot coupling region is neglected and propagation constants for orthogonal polarization states are considered degenerate. Thus, the three matrices $S$, $C$, and $L$ are rewritten as [19-21]

$$S=\left[ \begin{matrix} \exp\left( -i\beta_{0}L_{k} \right) & 0 \\ 0 & \exp\left( -i\beta_{0}L_{k} \right) \end{matrix} \right]\left[ \begin{matrix} s_{xx} & s_{xy} \\ -s_{xy} & s_{yy} \end{matrix} \right]$$

$$C=\left[ \begin{matrix} \exp\left( -i\beta_{0}L_{k} \right) & 0 \\ 0 & \exp\left( -i\beta_{0}L_{k} \right) \end{matrix} \right]\left[ \begin{matrix} c_{xx} & c_{xy} \\ -c_{xy} & c_{yy} \end{matrix} \right]$$

$$L=\left[ \begin{matrix} \alpha_{x} & 0 \\ 0 & \alpha_{y} \end{matrix} \right]\exp\left( -i\beta_{0}L_{r} \right)$$

(S12)

where $\beta_{0}$ is the effective propagation constant for both orthogonal polarization states, in both knot coupling region and ring section. Here the $\alpha_{x}$ and $\alpha_{y}$ represents the loss of the ring section, The ($s_{ij}, i,j=x,y$) denote self-coupling parameters and ($c_{ij}, i,j=x,y$) denote cross-coupling parameters, given as [21, 22]

$$s_{xx}=\cos\left( \kappa_{0}L_{k} \right)\cos\left( \kappa_{a}L_{k} \right)-\cos\varphi\sin\left( \kappa_{0}L_{k} \right)\sin\left( \kappa_{a}L_{k} \right)$$

$$s_{xy}=\sin\varphi\sin\left( \kappa_{0}L_{k} \right)\cos\left( \kappa_{a}L_{k} \right)$$

$$s_{yy}=\cos\left( \kappa_{0}L_{k} \right)\cos\left( \kappa_{a}L_{k} \right)+\cos\varphi\sin\left( \kappa_{0}L_{k} \right)\sin\left( \kappa_{a}L_{k} \right)$$

$$c_{xx}=\cos\left( \kappa_{0}L_{k} \right)\sin\left( \kappa_{a}L_{k} \right)+\cos\varphi\sin\left( \kappa_{0}L_{k} \right)\cos\left( \kappa_{a}L_{k} \right)$$

$$c_{xy}=\sin\varphi\sin\left( \kappa_{0}L_{k} \right)\sin\left( \kappa_{a}L_{k} \right)$$

$$c_{yy}=\cos\left( \kappa_{0}L_{k} \right)\sin\left( \kappa_{a}L_{k} \right)-\cos\varphi\sin\left( \kappa_{0}L_{k} \right)\cos\left( \kappa_{a}L_{k} \right)$$

$$\kappa_{a}=\frac{\kappa_{x}+\kappa_{y}}{2}, \kappa_{b}=\frac{\kappa_{x}-\kappa_{y}}{2}, \kappa_{0}=\sqrt{\kappa_{b}^{2}+\sigma^{2}}$$

$$\cos\varphi=\frac{\kappa_{b}}{\kappa_{0}}, \sigma=\frac{\theta}{L_{k}}$$

(S13)

where $\kappa_{x}$ and $\kappa_{y}$ are the deviated coupling coefficients for the two eigenstates of polarization, $\theta$ denotes the twisting angle of the knot area and $\sigma$ represents the twisting ratio.

If we simulate the polarization response of MKR based on this numerical model, we will find that the orthogonal linear polarization response distribution cannot be theoretically restored. As for the non-orthogonal (same-direction) response distribution, the slight yet persistent alternating intensities of resonant peaks also remain unexplained. Below, we will analyze this using typical numerical results as an example.

We first assume the condition where the coupling coefficients for both eigenstates of polarization are degenerate, i.e. the $\kappa_{b}=0 {\mu m}^{-1}$. Here $\kappa_{a}=15.7\times{10}^{-3} {\mu m}^{-1}$. The over-all length of cavity ${L_{k}+L}_{r}=7.5 mm$ with knot coupling area length of $L_{k}=400 \mu m$. $\alpha_{x}=\alpha_{y}=0.999$ in simulation. **Fig. S9a** shows the simulated resonance spectrum of MKR based on traditional model, where the incident light polarization angles are 15°, 45°, and 75°, respectively. As can be seen, no mode-splitting can be observed in the results, and the transmission spectrum remains unchanged, not affected by the incident polarization angle. As seen in **Fig. S9b**, the resonance depth of the only mode exhibits constant with respect to the input polarization angles. The Q-factor of this mode also shows bare variations with respect to the polarization angle of the incident light (**Fig. S9c**).


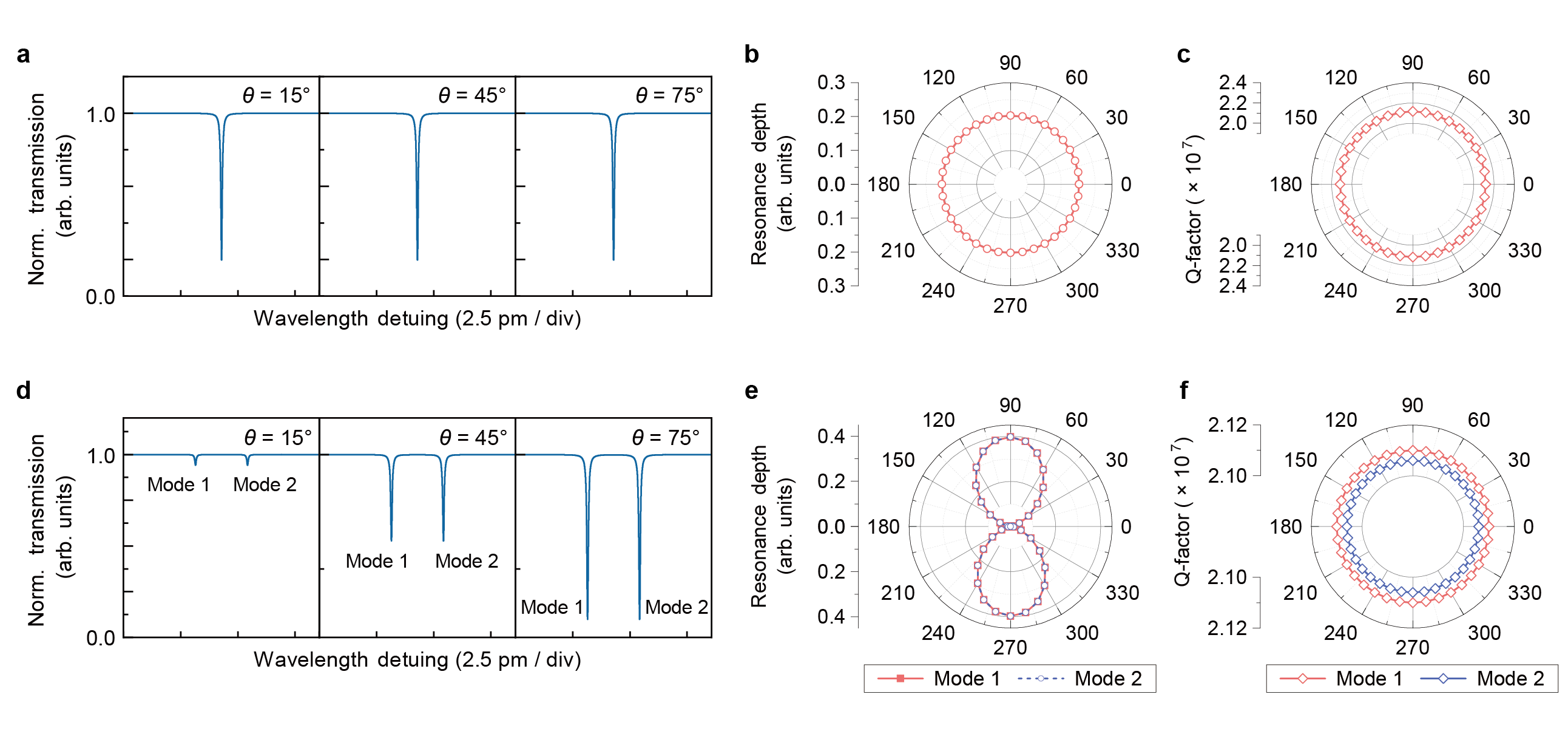


**Figure S9. Simulated polarization-dependent transmission of UHQ-MKR based on traditional model. a** Simulated transmission spectrum of UHQ-MKR with varied input polarization, where the coupling coefficients for both eigenstates of polarization are degenerate, with **b** the corresponding simulated resonance depth distribution versus input polarization angle and **c** the Q-factor distribution versus input polarization angle. **d** Simulated transmission spectrum of UHQ-MKR with varied input polarization, where the coupling coefficients for both eigenstates of polarization are different, with **e** the corresponding simulated resonance depth distribution versus input polarization angle for both modes and **f** the Q-factor distribution versus input polarization angle.

If one wants to achieve mode-splitting results with this traditional model, then the parameter $\kappa_{0}$ should not be 0, i.e. $\kappa_{b}\neq0$ or $\sigma\neq0$. Since they all contribute to the $\kappa_{0}$, we take the condition $\kappa_{b}\neq0$ for demonstration. Here we set the $\kappa_{b}=1.57\times{10}^{-3} {\mu m}^{-1}$, while other parameters remain unchanged. **Fig. S9d** shows the resonance spectrum under such circumstance, where the resonant modes split significantly, with incident light polarization angle of 15°, 45°, and 75°, respectively. However, as seen in **Fig. S9e**, the resonance depths of the two modes always stay the same, and change simultaneously with the input polarization angles, showing no alternating. This is because in the traditional model where birefringence is neglected, the splitting is not owed to the separation of both polarization states, but to the strong coupling between both states back and forth in the twisted region, which provides several possible paths for each polarization state. The phase shift difference between those possible paths induces the fine structure [21]. This makes the output of specific polarization state a superposition of both input polarization states, so the splitting modes change simultaneously with the incident polarization angle. The Q-factors of both modes are also independent of the polarization states (**Fig. S9f**). Therefore, the analysis based on the traditional numerical model cannot effectively reflect the phenomena observed in our experiments, which is precisely the motivation behind our improvement of this model.

Although the ring section of MKR is cylindrically symmetrical, the adjoined knot coupling region, which constitutes quite a part of the resonator, is heavily asymmetrical and capable of enabling birefringent guide-wave modes. Different from the previous traditional model, here in our model the birefringence in knot coupling region is taken into consideration, the three matrices $S$, $C$, and $L$ are rewritten as

$$S=\left[ \begin{matrix} \exp\left( -i\beta_{x}L_{k} \right) & 0 \\ 0 & \exp\left( -i\beta_{y}L_{k} \right) \end{matrix} \right]\left[ \begin{matrix} s_{xx} & s_{xy} \\ -s_{xy} & s_{yy} \end{matrix} \right]$$

$$C=\left[ \begin{matrix} \exp\left( -i\beta_{x}L_{k} \right) & 0 \\ 0 & \exp\left( -i\beta_{y}L_{k} \right) \end{matrix} \right]\left[ \begin{matrix} c_{xx} & c_{xy} \\ -c_{xy} & c_{yy} \end{matrix} \right]$$

$$L=\left[ \begin{matrix} \alpha_{x} & 0 \\ 0 & \alpha_{y} \end{matrix} \right]\exp\left( -i\beta_{0}L_{r} \right)$$

(S14)

where $\beta_{x}$ and $\beta_{y}$ are the effective propagation constant in knot coupling region for orthogonal polarization states respectively, and $\beta_{0}$ is the degenerate propagation constant in ring section. The birefringence value $B$ can be derived with $B=\left| \beta_{x}-\beta_{y} \right|/k_{0}$. $\alpha_{x}$ and $\alpha_{y}$ still represents the loss of the ring section, which are adopted as $\alpha_{x}=\alpha_{y}=0.999$ in simulation. The expressions of self-coupling parameters ($s_{ij}, i,j=x,y$) and cross-coupling parameters ($c_{ij}, i,j=x,y$) remain unchanged. $\theta$ is adopted as $2\pi$ in simulation.

Based on the birefringent model, varied polarization-dependent transmission features can be interpreted, as demonstrated in main manuscript.

**Supplementary Note 8: Advantages of knot-type microfiber resonators over loop and coil structures**

Microfiber-based resonators generally fall into three structural categories: loop, coil, and knot configurations. The knot-type resonator (MKR) adopted in this work offers significant advantages over the other two in terms of structural stability, coupling efficiency, and Q-factor performance, making it particularly suitable for applications that require ultra-high-quality factors.

The primary advantage of the knot resonator lies in its highly stable and easily reproducible coupling structure. The knot forms a compact, self-sustaining, tension-maintained coupling region that can maintain an efficient micron-scale gap for optical coupling even without any external support platform. This mechanically-constructed, intrinsically-aligned coupler minimizes perturbation-induced energy leakage and coupling asymmetry, enabling Q-factors on the order of 10⁷ even under standard laboratory conditions. In our experiments, multiple UHQ-MKR samples exhibited stable spectral responses with high Q and low loss, confirming the excellent repeatability and practical viability of this structure.

The microfiber loop resonator (MLR) is formed by twisting the tapered region of the microfiber into a loop. Coupling occurs near the contact point due to electrostatic or van der Waals forces [23]. As the input light passes through this region, part of the optical power couples into the loop and circulates within it, while the remaining power is transferred through evanescent coupling to the output fiber on the opposite side. Although the loop structure is simple, maintaining a stable coupling region is challenging. The coupling strength primarily depends on the proximity between two fiber segments, which lacks mechanical constraint and is thus susceptible to external vibration, thermal fluctuations, or operational inaccuracies. All of the above factors can degrade the Q-factor.

The microfiber coil resonator (MCR) is constructed by wrapping the microfiber around a supporting rod. When the spacing between adjacent coils is small, evanescent coupling occurs between turns[24]. By adjusting the number of turns and the inter-turn spacing, the coupling strength can be tuned. However, coil resonators face more severe structural challenges. While the long optical path theoretically supports denser mode distributions and extended light-matter interaction, it also introduces higher bending loss and increased modal interference [25]. Furthermore, the coupling control depends on manual positioning or mechanical fixtures, which lack the intrinsic precision of the knot structure [26]. Achieving sub-micron alignment is difficult, and the presence of supporting structures can introduce additional scattering losses, thereby lowering the Q-factor.

Still, we acknowledge that both loop and coil resonators hold theoretical potential for achieving high-Q performance. With precise control over coupling distances, minimization of packaging-induced loss, and further optimization of the coupling region design, these structures might also overcome current Q-factor limitations in the future. That said, based on current experimental realizations, the knot-type resonator stands out for its structural simplicity, highly stable coupling, and ability to achieve efficient resonance without complex alignment or supporting systems. These features make it the most advantageous microfiber resonator configuration for achieving high-Q performance, with application potential that is difficult to match with alternative designs.

Supplementary Note 9: Fiber laser based on UHQ-MKR


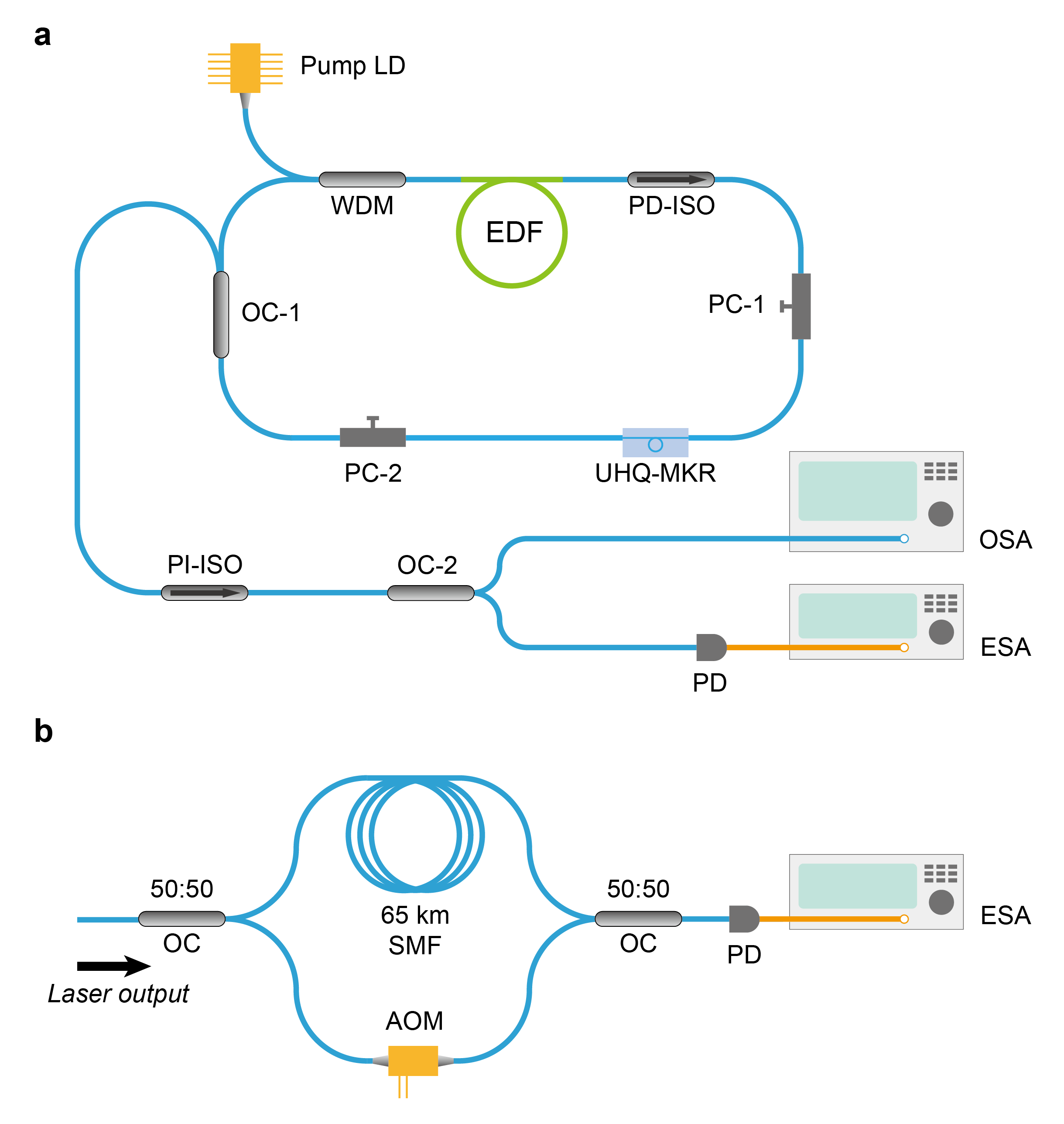


**Figure S10. Experimental setup of UHQ-MKR based fiber laser. a** Schematic diagram of all-fiber ring laser and measurement system. LD, laser diode; WDM, wavelength division multiplexer; EDF, erbium-doped fiber; PD-ISO, polarization-dependent isolator; PC, polarization controller; OC, optical coupler; PI-ISO, polarization-independent isolator; OSA, optical spectrum analyzer; PD, photodetector; ESA, electrical spectrum analyzer. **b** Schematic diagram of the delayed self-heterodyne measurement system. AOM, acoustic optical modulator.

The experimental setup of fiber laser based on UHQ-MKR is shown in **Fig. S10a**. The single loop ring fiber laser cavity is formed by one segment gain fiber and series of passive fiber devices. The heavily doped erbium-doped fiber (EDF, Er-110 4/125, Liekki) provides relatively large gain within short length (~0.5 m in experiments) and features a normal dispersion of -10.55 ps nm^-1^ km^-1^. The fibers comprising the remaining ring cavity are all single-mode fibers (SMF) except for a short portion of WDM’s pigtail, which is HI1060 fiber. A polarization-dependent isolator (PD-ISO) is employed to enforce unidirectional circulation and excite linear polarization states. Two polarization controllers (PCs) are employed to tune intracavity polarization states. The 90:10 optical coupler (OC) works as the output port where 10% of the oscillation power gets extracted from the cavity for monitoring. The output signal is transmitted to an optical spectrum analyzer (OSA) for optical spectrum characterization. By introducing the laser output directly into the photodetector (PD) connected with radio-frequency (RF) electrical spectrum analyzer (ESA), multi-longitudinal-mode-induced beating notes that may exist are monitored.

For obtaining the laser linewidth with delayed self-heterodyne measurement, the laser output should be introduced into a Mach-Zehnder interferometer as shown in **Fig. S10b** for beating. An 80 MHz acoustic optical modulator (AOM) and 65 km long SMF are embedded in two arms respectively. A 12 GHz high-speed photodetector (PD) connected with 26.5 GHz electrical frequency spectrum analyzer (ESA) gets employed for analysis of beating notes.

According to Fermi’s golden rule, in a laser system, the probability of transition and lasing at specific frequency depends on the density of states, and in a resonator the density of states can be enhanced by the Purcell factor

$$F_{P}=\left( 3\lambda^{3}Q \right)/\left( 4\pi^{2}V \right)$$

(S15)

where $V$ is the mode volume [27]. Since the UHQ-MKR has a mode volume much smaller than the laser cavity, its resonant frequencies have a higher density of states, leading to a higher transition probability and a lower laser threshold [28]. When the polarization state of the incident light is adjusted to the appropriate position, the high-Q modes can be effectively excited, thereby enabling laser output at the resonant modes.


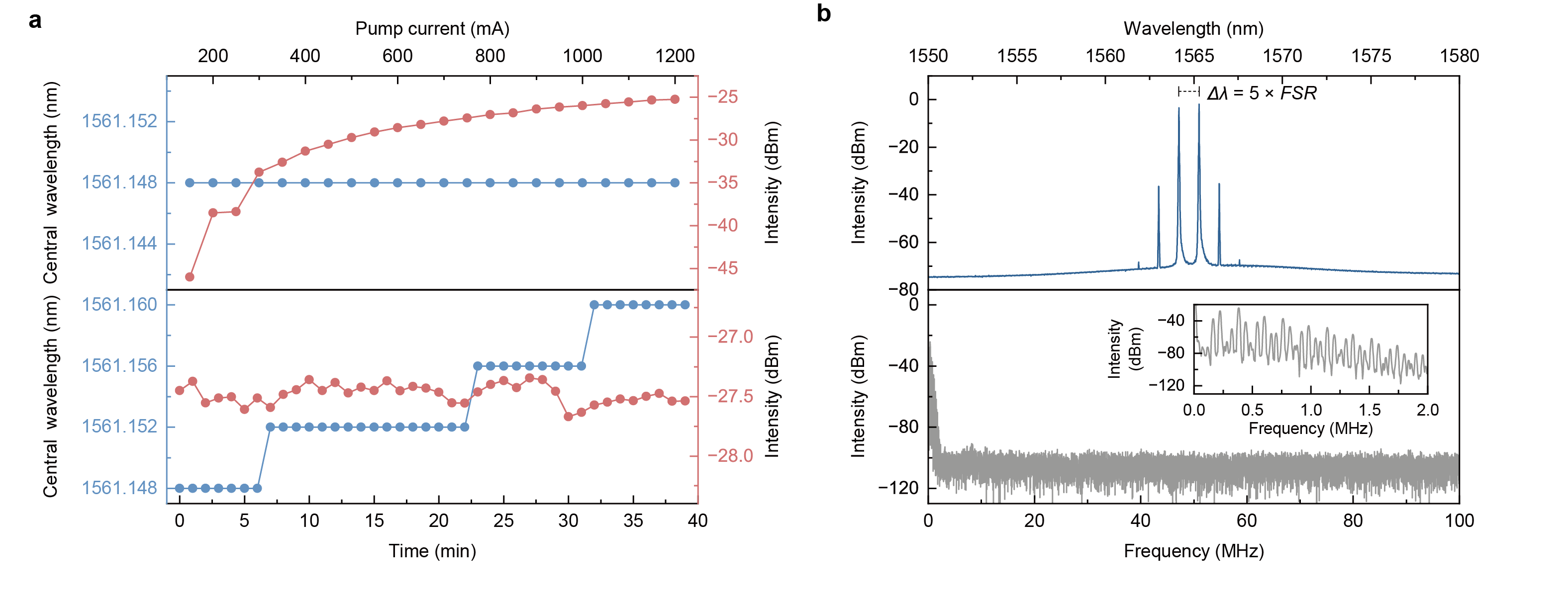


**Figure S11. Single-frequency and four-wave-mixing operation of UHQ-MKR based fiber laser. a** (top panel) Variation of laser central wavelength (left blue axis) and corresponding peak intensity (right red axis) with the pump current. (lower panel) Variation of laser central wavelength (left blue axis) and corresponding peak intensity (right red axis) with the operating time. **d** (top panel) Optical spectrum of the four-wave-mixing laser output. (lower panel) Corresponding RF spectrum within 100 MHz range, inset shows local scanning within 2 MHz range.

The relationships of the lasing wavelength and peak intensity variations versus the pump current are depicted in the top panel of the **Fig. S11a**. With the increase of the pump current from 150 mA to 1200 mA, the central wavelength shows no evident fluctuation within the acquisition resolution of OSA. The peak intensity of the single-frequency output shows an increasing trend from -46 to -25 dBm with the increase of pump current. Even at the small pump of 150 mA, the laser peak intensity can still ensure OSNR > 40 dB. For investigation of temporal stability of single-frequency operation, the output optical spectra are repeatedly scanned by OSA at 1 min intervals in time span of 40 min, as shown in the lower panel of **Fig. S11a**. Under the limited OSA resolution, the measured wavelength shows bare fluctuations within several minutes, but exhibiting observable red-shifting in the 40-min span due to the thermal effects arising from the heat accumulation in the UHQ-MKR. The corresponding power fluctuation shows a deviation of 0.08 dBm, demonstrating stable laser operation.

By further increasing the laser gain, degenerate and non-degenerate FWM processes occur within the UHQ-MKR, leading to parametric oscillation. The central frequency and newly generated sidebands are amplified by the fiber laser and then reinjected into the MKR, realizing a self-injected optical parametric oscillation process without the need for external detuning scans. As shown in the top panel of **Fig. S11b**, the primary comb generated exhibits a line spacing of 5 times the FSR, determined by the modulation instability gain spectrum depending on the microfiber’s dispersion and nonlinearity. At this stage, the OSNRs of the two central comb teeth both exceed 70 dB, demonstrating high stability. The lower panel of **Fig. S11b** shows the RF spectrum of the laser under this regime. Here, it is evident that supermode instability caused by multi-longitudinal-mode oscillation remains effectively suppressed. However, due to the relatively high intracavity power, the erbium-doped fiber exhibits gain saturation modulation, resulting in random beating components within the 0~2 MHz range. It is believed that integration of MKR devices with higher Q-factors and optimization of laser parameters could enable broader-band comb or even soliton-regime comb generation, offering an all-fiber solution for microresonator comb source development.

Reference

1. Brambilla, G., Finazzi, V. & Richardson, D. J. Ultra-low-loss optical fiber nanotapers. *Optics Express* **12**, 2258-2263 (2004).

2. Leon-Saval, S. G. et al. Supercontinuum generation in submicron fibre waveguides. *Optics Express* **12**, 2864-2869 (2004).

3. Sumetsky, M., Dulashko, Y. & Hale, A. Fabrication and study of bent and coiled free silica nanowires: self-coupling microloop optical interferometer. *Optics Express* **12**, 3521-3531 (2004).

4. Tong, L. M. et al. Subwavelength-diameter silica wires for low-loss optical wave guiding. *Nature* **426**, 816-819 (2003).

5. Sumetsky, M. et al. Thinnest optical waveguide: experimental test. *Optics Letters* **32**, 754-756 (2007).

6. Brambilla, G. et al. Optical fiber nanowires and microwires: fabrication and applications. *Advances in Optics and Photonics* **1**, 107-161 (2009).

7. Rasoloniaina, A. et al. Controling the coupling properties of active ultrahigh-*Q* WGM microcavities from undercoupling to selective amplification. *Scientific Reports* **4**, 4023 (2014).

8. Mei, Y. L. et al. Whispering gallery mode microsphere resonator based on cylindrical air cavity coupling. *Optics Letters* **48**, 1594-1597 (2023).

9. Yang, Y. et al. Recent progress of in-fiber WGM microsphere resonator. *Frontiers of Optoelectronics* **16**, 10 (2023).

10. Hoffman, J. E. et al. Ultrahigh transmission optical nanofibers. *AIP Advances* **4**, 067124 (2014).

11. Zhang, J. et al. Optical microfiber or nanofiber: a miniature fiber-optic platform for nanophotonics. *Photonics Insights* **3**, R02 (2024).

12. Brambilla, G., Xu, F. & Feng, X. Fabrication of optical fibre nanowires and their optical and mechanical characterisation. *Electronics Letters* **42**, 517-519 (2006).

13. Schermer, R. T. & Cole, J. H. Improved bend loss formula verified for optical fiber by simulation and experiment. *IEEE Joural of Quantum Electronics* **43**, 899-909 (2007).

14. Velamuri, A. V. et al. Investigation of planar and helical bend losses in single- and few-mode optical fibers. *Journal of Lightwave Technology* **37**, 3544-3556 (2019).

15. Vienne, G., Li, Y. H. & Tong, L. M. Microfiber resonator in polymer matrix. *IEICE Transactions on Electronics* **E90C**, 415-421 (2007).

16. Okamoto, K. Fundamentals of Optical Waveguides (Academic Press, 2006).

17. Michalske, T. A. & Freiman, S. W. A molecular mechanism for stress corrosion in vitreous silica. *Journal of the American Ceramic Society* **66**, 284-288 (1983).

18. Muraoka, M., Ebata, K. & Abe, H. Effect of humidity on small-crack growth in silica optical fibers. *Journal of the American Ceramic Society* **76**, 1545-1550 (1993).

19. Liao, Y. P. et al. Resonant mode characteristics of microfiber knot-type ring resonator and Its salinity sensing experiment. *IEEE Photonics Journal* **7**, 6802308 (2015).

20. Wang, G. H. et al. The numerical modeling of 3D microfiber couplers and resonators. *IEEE Photonics Technology Letters* **28**, 1707-1710 (2016).

21. Wang, G. H. et al. Polarization effects in microfiber loop and knot resonators. *IEEE Photonics Technology Letters* **22**, 586-588 (2010).

22. Morishita, K. & Yamaguchi, T. Wavelength tunability and polarization characteristics of twisted polarization beamsplitting single-mode fiber couplers. *Journal of Lightwave Technology* **19**, 732-738 (2001).

23. Sumetsky, M. et al. Optical microfiber loop resonator. *Applied Physics Letters* **86**, 161108 (2005).

24. Jung, Y. et al. Embedded optical microfiber coil resonator with enhanced High-*Q*. *IEEE Photonics Technology Letters* **22**, 1638-1640 (2010).

25. Xu, F. & Brambilla, G. Manufacture of 3-D microfiber coil resonators. *IEEE Photonics Technology Letters* **19**, 1481-1483 (2007).

26. Jiang, X. et al. Demonstration of optical microfiber knot resonators. *Applied Physics Letters* **88**, 223501 (2006).

27. Purcell, E. M. in Confined Electrons and Photons NATO ASI Series, vol. 340 (eds Burstein, E. & Weisbuch, C.) Ch. 40 (Springer, 1995).

28. Sandoghdar, V. et al. Very low threshold whispering-gallery-mode microsphere laser. *Physical Review A* **54**, R1777-R1780 (1996).
